# The expansion of the TRB and TRG genes in domestic goats (*Capra hircus)* is characteristic of the ruminant species

**DOI:** 10.1186/s12864-020-07022-x

**Published:** 2020-09-11

**Authors:** Francesco Giannico, Serafina Massari, Anna Caputi Jambrenghi, Adriano Soriano, Angela Pala, Giovanna Linguiti, Salvatrice Ciccarese, Rachele Antonacci

**Affiliations:** 1 Department of Veterinary Medicine, University of Bari “Aldo Moro”, Bari, Italy; 2 Department of Biological and Environmental Science and Technologies, University of Salento, Lecce, Italy; 3 Department of Agricultural and Environmental Science, University of Bari “Aldo Moro”, Bari, Italy; 4 Department of Biology, University of Bari “Aldo Moro”, 70124 Bari, Italy

**Keywords:** T cell receptor, TRB locus, TRG locus, TRB genes, TRG genes, Goat genome, Ruminants, ImMunoGeneTics database

## Abstract

**Background:**

Goats (*Capra hircus*), one of the first domesticated species, are economically important for milk and meat production, and their broad geographical distribution reflects their successful adaptation to diverse environmental conditions. Despite the relevance of this species, the genetic research on the goat traits is limited compared to other domestic species. Thanks to the latest goat reference genomic sequence (ARS1), which is considered to be one of the most continuous assemblies in livestock, we deduced the genomic structure of the T cell receptor beta (TRB) and gamma (TRG) loci in this ruminant species.

**Results:**

Our analyses revealed that although the organization of the goat TRB locus is broadly similar to that of the other artiodactyl species, with three in-tandem D-J-C clusters located at the 3′ end, a complex and extensive series of duplications have occurred in the *V* genes at the 5′ end, leading to a marked expansion in the number of the *TRBV* genes. This phenomenon appears to be a feature of the ruminant lineage since similar gene expansions have also occurred in sheep and cattle.

Likewise, the general organization of the goat *TRG* genes is typical of ruminant species studied so far, with two paralogous TRG loci, TRG1 and TRG2, located in two distinct and distant positions on the same chromosome as result of a split in the ancestral locus. Each TRG locus consists of reiterated V-J-J-C cassettes, with the goat TRG2 containing an additional cassette relative to the corresponding sheep and cattle loci.

**Conclusions:**

Taken together, these findings demonstrate that strong evolutionary pressures in the ruminant lineage have selected for the development of enlarged sets of *TRB* and *TRG* genes that contribute to a diverse T cell receptor repertoire. However, differences observed among the goat, sheep and cattle *TRB* and *TRG* genes indicate that distinct evolutionary histories, with independent expansions and/or contractions, have also affected each ruminant species.

## Background

Ruminants belong to the order Cetartiodactyla, which comprises even-toed ungulates and cetaceans. Six families are included within the ruminant suborder, among which the family Bovidae consists of approximately 143 species and includes most of the economically important livestock species, such as cattle, sheep, and goat.

Ruminant species are the most efficient herbivores and are distributed across diverse territories in terms of latitude (from tropical to arctic regions), altitude (from plains to plateaus) and habitat (from deserts to rainforests). Beyond their adaptability, these species also show recognizable divergent phenotypes, including distinct morphological characteristics in the headgear (e.g., ossicones in giraffids, antlers in cervids and horns in bovids) [1] and variations in body size, which ranges from 2 kg (lesser deer) to 1200 Kg (giraffa and buffalo) [2].

The recent release of complete genome assemblies has enabled investigation of the genetic basis and evolution of complex traits in several ruminant species [3]. Among other findings, studies have shown that the ruminant genome has unique characteristics in terms of the evolution and diversification of gene families. For instance, it is known that ruminants have a more efficient digestion system than other herbivores due to the presence of a four-chambered stomach in which the largest compartment, the rumen, utilizes microbial flora to digest plant materials. It was found that the recruitment of lysozyme C to a digestive role is associated with an expansion in the size of the lysozyme C gene family. In fact, the ruminant species have 10 or more *lysozyme C* genes, while most mammals have only one or a few *lysozyme C* genes [4, 5].

Other gene families in which a gene expansions have been observed in ruminants, include those encoding for the α, β, γ and δ chains of the αβ or γδ T cell receptor (TR). Each gene family is organized in a distinct genomic locus that are named TRA, TRB, TRG or TRD based on the different TR chain. To obtain an exhaustive repertoire of TR, each gene family encodes the variable domain of its own chain through a random process of DNA rearrangement that involves somatic recombination of *V* (*variable*), *D* (*diversity*) and *J* (*joining*) genes for the β and δ chains, and *V* and *J* genes for the α and γ chains. After transcription, the resulting rearranged V(D) J region, is spliced to the *C* (*constant*) gene that encodes the constant domain of the receptor. The resulting chain is a protein with the variable domain composed of seven distinguishable regions: three hypervariable loops or complementarity-determining regions (CDR-IMGT) and four framework regions (FR-IMGT). Two of the CDR loops, CDR1-IMGT and CDR2-IMGT, are encoded by the *V* gene. The third CDR loop (CDR3-IMGT) reflects the ability of the *V* gene to rearrange to any (*D*) *J* gene [6]. The *V*, *D*, *J* and *C* genes exist within each TR locus as multigene subfamilies, and the total number of their gene members varies in different species. Thus, identifying the TR germline repertoire within a given species provides information about its potential for mounting an adaptative immune response against antigens.

The genomic organization of the TRB locus, which encodes the TR β chain, has been determined in representative species of several mammalian orders. The general genomic structure consists of a semi-cluster organization, with a pool of *TRBV* genes, positioned upstream of in-tandem aligned TRBD-J-C clusters, each of which is composed of a single *TRBD* gene, several *TRBJ* genes and one *TRBC* gene. An inverted *TRBV* gene lies at the 3′ end of the last *TRBC* gene. In human, mouse, and several other mammals (https://www.imgt.org/IMGTrepertoire/: *Homo sapiens*, *Mus musculus* [7–11];), two TRBD-J-C clusters have been identified. In contrast, in the artiodactyl lineage [12–17], a duplication event within the 3′ end of the TRB locus has led to the generation of a third TRBD-J-C cluster. Moreover, ruminants appear to have an increased number of genes in the TRBV gene pool, which is the largest in cattle (134 *TRBV* genes) [13], and sheep (94 *TRBV* genes) [11] and resulted from massive expansion of mainly two TRBV gene subgroups.

Similar findings have been reported for the sheep TRAV and TRDV germline repertoire as well. In this case, the high number of *TRAV* and *TRDV* genes is explained by the multigene nature of several TRAV subgroups and the gene expansion of the TRDV1 subgroup [18, 19]. Such expansion of the TRAV and TRDV gene repertoires also appears in the bovine genome [20–22].

However, the most evident difference between ruminants (i.e., cattle and sheep) and other mammalian species, including other artiodactyls, regards the genomic organization of the TRG locus encoding the TR γ chain [23]. In ruminants, this locus is organized into several functional in-tandem V-J-J-C clusters or cassettes which originated from repeated duplications of an ancestral cluster [24–26] and split into two distinct chromosomal positions to generate two loci [27, 28]. One locus, named TRG1, is located within a region of homology with other mammalian species, whereas the other locus, TRG2, is not included in the region of synteny and thus appears to be unique to ruminants.

Goat, a species closely related to sheep and cattle, diverged from sheep approximately 15 Mya [29] and from cattle approximately 30 Mya [30]. Despite the economic importance of goats, little is known about their TR loci.

Thanks to the availability of a new genomic resource, in the present study, we characterized the genomic organization of the goat TRB and TRG loci and compared them to what is presently known regarding their organization in related species. Our results provide new insights into ruminant diversity and evolution.

## Results

### Genomic organization of the TRB and TRG loci

The current highly contiguous reference genome ARS1 (BioProject ID: PRJNA290100) was used to identify the TRB and the TRG loci in goats (*Capra hircus*). Pre-existing data on physical mapping assigned these loci to goat chromosome 4 [27, 31]. Thus, using the corresponding sheep genomic sequences as reference, we retrieved from the *Capra hircus* chromosome 4 genomic scaffold (GenBank ID: CM004565) the TRB and TRG genomic regions and established their structures.

### The TRB locus

**Fig. 1:**
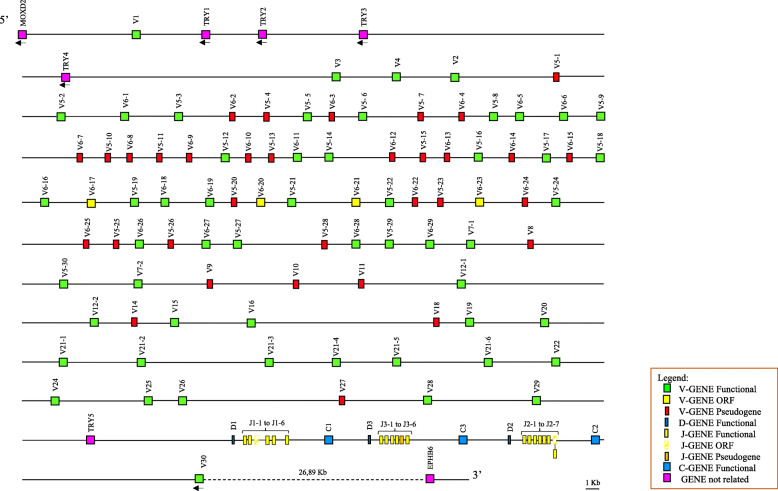
Schematic representation of the genomic organization of the goat TRB locus deduced from the genome assembly ARS1. The diagram shows the position of all the related and unrelated TRB genes according to nomenclature. The boxes representing the genes are not to scale. The exons are not shown. The arrows indicate the transcriptional orientation of the genes. The TRBJ2–7 gene functional found in the cDNA clones is also indicated (see Results)

To determine the genomic organization of the goat TRB locus, a sequence approximately 560 kb in length was retrieved from the whole chromosome 4 contig, comprising the IMGT 5′ borne (*MOXD2* gene) and the IMGT 3′ borne (*EPHB6* gene) that flank all mammalian TRB loci studied to date. We identified and annotated all *TRB* genes using the sheep sequence as a reference [11, 12]. The general structural organization of the goat TRB locus is conserved with respect to the other artiodactyl species [11–14, 16, 17], with a library of *TRBV* genes positioned at the 5′ end of three D-J-C clusters, that is followed by a single *TRBV* gene located at the 3′ end in an inverted transcriptional orientation (Fig. 1). Moreover, we identified and annotated the *trypsin-like serine protease* (*TRY*) genes that are typically interspersed among the mammalian *TRB* genes. Four *TRY* genes are located downstream of *TRBV1*, and one *TRY* gene is located upstream of the D-J-C region (Fig. 1). The classification, position and predicted functionality of all related and unrelated *TRB* genes are reported in Additional file 1: Table S1 and Additional file 2: Table S2, respectively.

### Classification and phylogenetic analysis of the *TRBV* genes

We annotated a pool of 91 *TRBV* genes grouped into 27 distinct subgroups according to the criterion that sequences with nucleotide identity above 75% belong to the same subgroup [32]. Five subgroups are multimembers with a massive expansion of the TRBV5 (30 genes), TRBV6 (29 genes) and TRBV21 (6 genes), while both the TRBV7 and TRBV12 subgroups consist of two genes. Out of the 91 *TRBV* genes, 54 (approximately 60%) are predicted to be functional genes as defined by the IMGT rules (see “Methods”), and 37 (40%) are not functional (pseudogenes and ORF) (Additional file 3: Table S3). The deduced amino acid sequences of the potential functional germline *TRBV* genes, ORF and in-frame pseudogenes are shown in Additional file 4: Figure S1, where they are aligned according to IMGT unique numbering for the V-REGION [33] to maximize the percentage of identity.

**Fig. 2:**
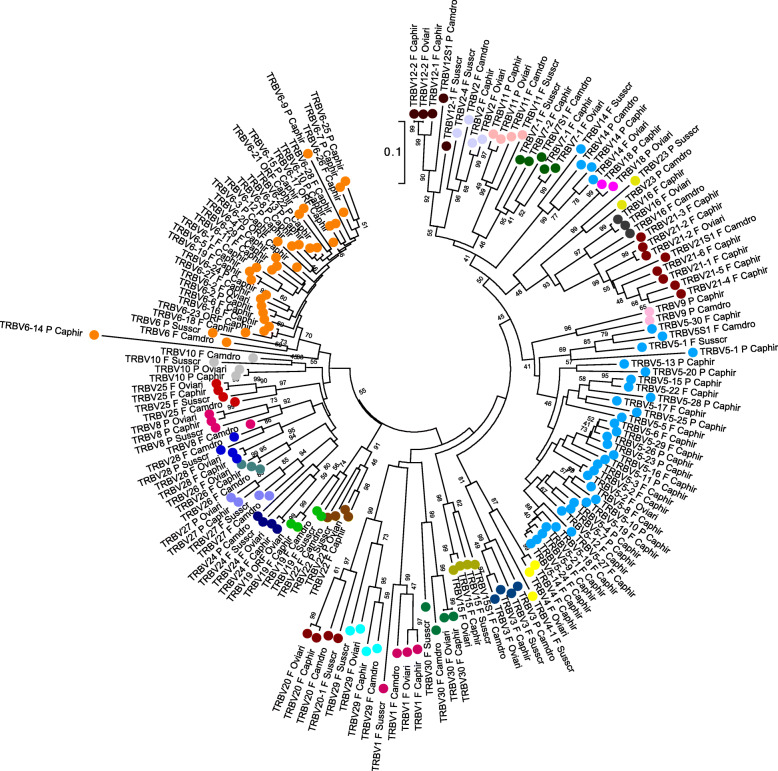
The NJ tree inferred from the goat, sheep, pig and dromedary TRBV gene sequences. The evolutionary analysis was conducted in MEGA7 [35]. The optimal tree with the sum of branch length = 12.78116203 is shown. The percentage of replicate trees in which the associated taxa clustered together in the bootstrap test (100 replicates) is shown next to the branches [36]. The tree is drawn to scale with branch lengths in the same units as those of the evolutionary distances used to infer phylogenetic trees. The evolutionary distances were computed using the p-distance method [37] and are in the units of the number of base differences per site. The analysis involved 166 nucleotide sequences. Codon positions included were 1st + 2nd + 3rd + Noncoding. All positions containing gaps and missing data were eliminated. There were a total of 92 positions in the final dataset. The goat TRBV subgroup classification is performed according to the clustering with the orthologous artiodactyl TRBV subgroups. The different colours highlight the distribution of the phylogenetic groups. The gene functionality according to IMGT rules (F: functional, ORF: open reading frame, P: pseudogene) is indicated. The IMGT 6-letter for species (Caphir, Oviari, Susscr and Camdro,) standardized abbreviation for taxon is used

To validate the classification and the membership of the goat *TRBV* genes to the subgroups, the evolutionary relationship of these genes was investigated in the context of the artiodactyl order by comparing all goat gene sequences with the corresponding sheep, pig and dromedary sequences, adopting two selection criteria: (i) only potential functional genes and in-frame pseudogenes were included, and (ii) only one gene was selected per subgroup. Thus, the V-REGION nucleotide sequences of all selected *TRBV* genes were combined in the same alignment, and an unrooted phylogenetic tree was constructed using the NJ method [34] (Fig. 2). The tree shows that each of the 27 goat subgroups forms a monophyletic group, when present, with corresponding sheep, pig and dromedary genes, consistent with the birth of distinct subgroups prior to the divergence of the different species.

Moreover, in accordance with the accepted phylogeny of these species, the goat and sheep orthologous genes are more closely related to each other than to their pig and dromedary counterparts in all groupings but one (*TRBV29*). As in sheep, the TRBV23 subgroup is missing in the goat locus. Hence, the phylogenetic clustering confirmed the classification of each goat TRBV subgroup as orthologous to the corresponding artiodactyl subgroup.

**Fig. 3:**
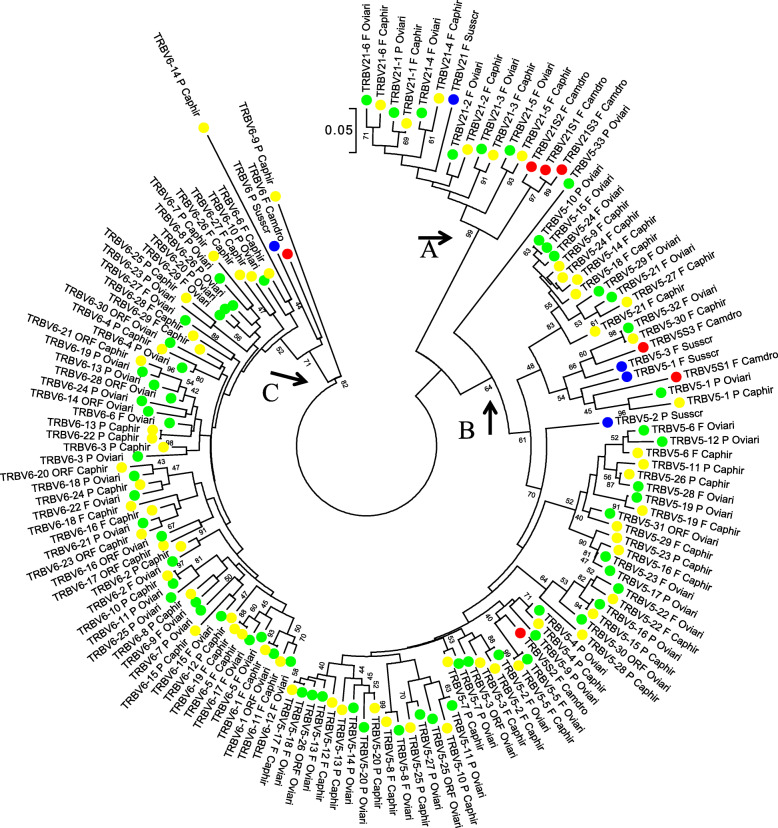
The NJ tree inferred from the goat, sheep, pig and dromedary TRBV5, TRBV6 and TRBV21 gene sequences. The evolutionary analysis was conducted in MEGA7 [35]. The optimal tree with the sum of branch length = 5.91955883 is shown. The percentage of replicate trees in which the associated taxa clustered together in the bootstrap test (100 replicates) is shown next to the branches [36]. The tree is drawn to scale with branch lengths in the same units as those of the evolutionary distances used to infer phylogenetic trees. The evolutionary distances were computed using the p-distance method [37] and are in the units of the number of base differences per site. The analysis involved 146 nucleotide sequences. Codon positions included were 1st + 2nd + 3rd + Noncoding. All positions containing gaps and missing data were eliminated. There were a total of 80 positions in the final dataset. The orthologous artiodactyl TRBV subgroups form three major monophyletic grouping: A (*TRBV21* genes), B (*TRBV5* genes) and C (*TRBV6* genes). The different colours highlight the genes within each species, yellow for goat, green for sheep, blue for pig and red for dromedary genes. The gene functionality according to IMGT rules (F: functional, ORF: open reading frame, P: pseudogene) is indicated. The IMGT 6-letter for species (Caphir, Oviari, Susscr and Camdro,) standardized abbreviation for taxon is used

A second phylogenetic tree was constructed to investigate the evolutionary origin of the goat multimember TRBV subgroups. Thus, the V-REGION nucleotide sequences of all goat TRBV5, TRBV6 and TRBV21 subgroup genes were compared with the sequences of all genes included in the corresponding sheep, pig and dromedary TRBV subgroups to obtain an NJ phylogenetic tree [34] (Fig. 3). The tree shows three major monophyletic groupings (A-C), corresponding to the three TRBV subgroups. Taking into account that the same subgroups are also expanded in the sheep locus, it is evident that within each grouping, goat and sheep genes intermingle. In particular, in branch A, each goat *TRBV21* gene is tightly related to a corresponding sheep gene, indicating the existence of a common ancestor of the two species in which the duplication events occurred. On the other hand, the grouped dromedary *TRBV21* genes appear to have evolved in a species-specific manner. Likewise, within branches B and C, very often a goat *TRBV5* or *TRBV*6 gene forms a monophyletic clade with a corresponding sheep gene (as shown by the alternated yellow and green circles in Fig. 3), indicating that most duplication events were shared between the two species, whereas a minor number of duplication events affected the goat and sheep lineages independently. However, no such striking gene duplications have affected swine or dromedary species*.*

### Genomic architecture of the TRBV-cluster

The goat TRBV region was aligned against itself with the PipMaker program to identify, through identity lines, the model of duplications that have generated the expansion of the TRBV gene subgroups (Additional file 5-6: Figure S2A-B). At first glance, an inspection of the dot-plot matrix reveals a background spread throughout the region probably due to intergenic sequences involved in the massive duplicative events of the *TRBV* genes.

However, the matrix confirms the high level of nucleotide identity between *TRBV* genes as indicated by dots and diagonal lines that correspond with gene location. In particular, we observed parallel lines forming two square shapes (shown in red in Additional file 5: Figure S2A) that identify multiple tandem duplications.

The first and wider square occupies an area of approximately 182 kb where the *TRBV5* and *TRBV6* genes have given rise to a complex series of tandem duplicative events (Additional file 6: Figure S2B). The tandem duplication seems to have involved a single *TRBV5* or *TRBV6* gene, or to consist of a duplicated TRBV5-TRBV6 unit. Furthermore, the presence of parallel lines of different sizes indicates the presence of higher order repetitive units, which include various arrangements (alternations) of the *TRBV5* and *TRBV*6 genes, such as the 4x(TRBV5-TRBV6) unit contained in the regions from *TRBV5–9* to *TRBV6–10*, from *TRBV5–13* to *TRBV6–14*, from *TRBV5–20* to *TRBV6–23* and from *TRBV5–14* to *TRBV6–15*, as indicated by longer parallel lines.

The other square shape identifies the tandem duplications of the TRBV21 subgroup genes (Additional file 5: Figure S2A).

### Description of the D-J-C region

The goat *TRBD*, *TRBJ* and *TRBC* genes were distributed within three in-tandem D-J-C clusters positioned at the 3′ end of the TRB locus, with D-J-C cluster 3 positioned between D-J-C clusters 1 and 2 (Fig. 1).

The nucleotide and deduced amino acid sequences of the three *TRBD* genes identified in the region are shown in Additional file 7: Figure S3A. Their length is identical to the corresponding sheep genes, with the exception of the *TRBD1*, which has one fewer nucleotide in goat. The three *TRBD* genes consist of 13 bp (*TRBD1*), 17 bp (*TRBD3*) and 16 bp (*TRBD2*) G-rich stretches that can be productively read through their three coding phases, and they encode till 4 glycine residues, depending on the phase. The RS sequences that flank the 5′ and 3′ sides of the coding region are well conserved with respect to the consensus sequence.

The nucleotides and deduced amino acid sequences of all the *TRBJ* genes identified in the region are shown in Additional file 8: Figure S3B. The *TRBJ* genes were classified, based on the international nomenclature (IMGT®, http://www.imgt.org, [38, 39]), according to the subsequent *TRBC* gene and numbered in agreement with the corresponding sheep TRBJ clusters. All *TRBJ* genes are between 44 bp and 53 bp in length and most contain the canonical FGXG amino acid motif whose presence defines the functionality of the *J* genes. The exceptions were *TRBJ1–3* and *TRBJ3–6*, which lack the FGXG J-MOTIF. Each *TRBJ* gene is flanked by a 12 RS sequence at the 5′ end and by a donor splice site at the 3′ end. All the RSs are well conserved with respect to the consensus sequence, except for the non-canonical RS of *TRBJ3–6* and the *TRBJ1–3* and *TRBJ2–7* heptamers, where the TG located in the last two nucleotide positions is mutated to GC. The *TRBJ3–6* gene also contains a non-canonical donor-splice site.

Like the *TRBC* genes of all known mammalian species, the three goat *TRBC* genes are nearly identical and are composed of four exons and three introns. The goat *TRBC* genes encode a similar protein of 178 amino acids (AA). The difference of 16 nucleotides between them results in seven AA changes, with six clustered in the extracellular region (C-domain) and one in the cytoplasmic region (Additional file 9: Figure S3C). As in sheep, the C-domain encoded by exon (EX) 1 is 130 AA in length due to the identical length of the FG loop compared to the other artiodactyl species [14, 16]. The C region also comprises a connecting region of 21 AA (encoded by EX2 and the 5′ part of EX3) with a cysteine involved in interchain disulphide binding, a transmembrane region of 21 AA (encoded by the 3′ part of EX3 and the first codon of EX4) and a cytoplasmic region of 5 AA (encoded by EX4).

### Analysis of cDNAs

To assess the quality of the genome assembly used to characterize the goat TRB locus and to evaluate the in silico predicted functional competency of the genes, we analysed the goat cDNA clone dataset available at IMGT/LIGM-DB (http://www.imgt.org). Due to the absence of a characterized goat germline reference sequence, each cDNA sequence in the database was annotated with a provisional name, indicated by the letter S, that identifies the TRBV subgroups (“IMGT gene” in Additional file 10: Table S4). Hence, the comparison between germline and cDNA sequences allowed us to evaluate the participation of the germline *TRBV* and *TRBJ* genes in the generation of the expressed repertoire and to update the IMGT classification of the genes.

Only five cDNAs perfectly matched the corresponding germline TRBV genes sequences (Additional file 10: Table S4). Twenty expressed *TRBV* genes showed 97 to 99% nucleotide identity to their corresponding germline genes. We referred to these as alleles based on the assumption that sequences sharing > 97% of nucleotide identity represent the same gene [14, 16]. In contrast, the highest percentage of nucleotide identity between the *TRBV* gene sequences of two cDNA clones (TCRVB1 and TCRVB53 in Additional file 10: Table S4) and the TRBV5 and TRBV7 germline gene sequences, respectively, was < 97%, indicating that these expressed genes may represent products of additional genes absent in the current assembly, even if polymorphism conditions cannot be ruled out. However, the functional competency of 21 *TRBV* genes belonging to 17 subgroups was confirmed. Among them was the *TRBV3* gene, which has a stop-codon at position 108 (Additional file 4: Figure S1A) that disappears during somatic recombination, resulting in a productive transcript (TCRVB54 clone in Additional file 10: Table S4). As expected, no corresponding cDNAs were found for the germline *TRBV* genes classified as pseudogenes.

All the *TRBJ* genes were unambiguously classified with respect to their corresponding genomic sequences. A total of 10 out of 17 functional TRBJ sequences were detected. The TRBJ2 cluster is the most highly represented (19/27), while six clones retain a member of the TRBJ3 cluster and three clones retain the TRBJ1 gene set. Notably, *TRBJ2–7*, classified as an ORF for a non-canonical heptamer, was found to be rearranged in five different cDNA clones. Furthermore, all three *TRBC* genes were identified among the cDNAs, each spliced to the rearranged *TRBJ* of its own D-J-C cluster.

**Fig. 4:**
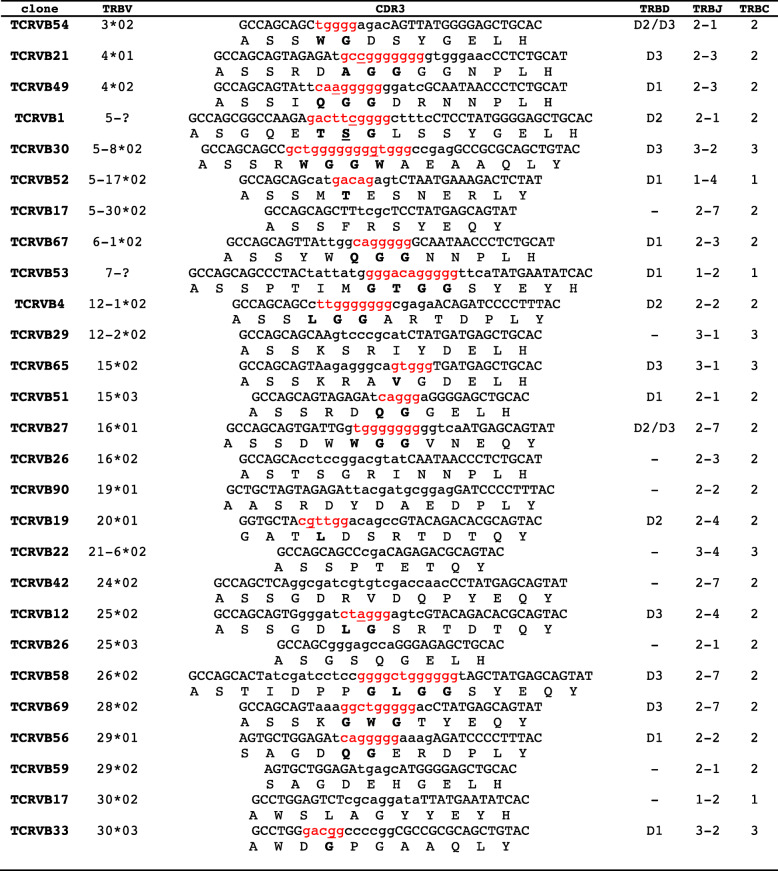
CDR3 nucleotide and predicted amino acid sequences retrieved from the TRB cDNA clones. CDR3-IMGT sequences are shown from codon 105 (the codon after the 2nd-CYS 104 of the V-REGION) to codon 117 (the codon before J-PHE 118 of the J-REGION) according to the unique numbering [33]. The CDR3 nucleotide/amino acid sequence, and the classification of the *TRBV* and *TRBJ* genes of each clone are also listed. The *TRBV* genes retained in TCRVB1 and TCRVB53 clones were assigned to the corresponding germline subgroup only (see text). Nucleotides of the 3’V-REGION and of the 5’J-REGION are indicated in uppercase letters. The sequences considered to present recognizable *TRBD* genes are indicated in red lowercase letters and the nucleotide substitutions are underlined. The amino acids belonging to a *TRBD* gene are in bold. Nucleotides that cannot be attributed to any V, D or J region (N-nucleotides), are indicated in lower cases on the left and on the right sides of the TRBD regions. The TRBC gene and the name of each clone are also reported

For the determination of the *TRBD* genes, the nucleotide sequences of the CDR3-IMGT of each expressed product, from codon 105 to 117 according to the unique numbering [33], were excised and analysed in detail (Fig. 4). The deduced amino acid sequences of the CDR3 loop reveal that it is heterogeneous in regard to amino acid composition and length without specific differences in relation to *TRBV* or *TRBJ* gene usage. The mean length of the CDR3 loop was 12.5 AA (with a range of 9 to 16 AA), which is in line with the previous analyses of sheep [40], pig [16] and dromedary [14] TRB transcripts.

Compared to genomic TRBD, the sequences located in the CDR3 regions were considered to belong to a *TRBD* gene if they constituted a stretch of at least five consecutive nucleotides*.* Under this criterion, the *TRBD* gene was unambiguously identified in 16 out of 27 sequences (59%), with *TRBD1* present in 7 clones, *TRBD3* in six clones and *TRBD2* in three clones. The remaining 11 sequences either did not have an identifiable *TRBD* gene (9 clones), or it was not possible to distinguish between *TRBD3* and *TRBD2* (2 clones) because of their similar germline sequences (Fig. 4). The absence of a TRBD region could be interpreted as the presence of a direct V-J junction. However, it is also possible that nucleotide trimming masked the initial participation of the *TRBD* gene during rearrangement. Four cDNA clones within the D region exhibited a nucleotide substitution with respect to the germline sequences.

Because the genomic organization of the D-J-C region is known, a formal interpretation of the D-J-C arrangement was also possible. Intra-cluster rearrangements are appreciable in seven clones, with three TRBD2-TRBJ2, two TRBD1-TRBJ1 and two TRBD3-TRBJ3 recombination. Nine rearrangements can be interpreted by direct 5′ to 3′ joining across the clusters (inter-cluster rearrangements), with four TRBD1-TRBJ2 and one TRBD1-TRBJ3, and four TRBD3-TRBJ2 recombinations.

### TRG1 and TRG2 genomic organization

The genomic structure of the goat TRG loci was determined with an approach similar to the one used to identify the TRB locus. Using the sheep TRG1 (GenBank accession number DQ992075) and TRG2 (GenBank accession number DQ992074) sequences as reference, we identified two distinct genomic regions on the *Capra hircus* chromosome 4 genomic scaffold. The first region (pos: c37490000–37,659,887) corresponds to the TRG1 locus, and the second (pos.: 70113000–70,256,000) is representative of the TRG2 locus, confirming previous FISH mapping data of two TRG regions in Bovidae (i.e., sheep, goat, cattle and river buffalo [27]). Moreover, the availability of the whole sequence of goat chromosome 4 has allowed us to measure the exact distance between the two paralogous TRG loci (approximately 32 Mb) and to realize that they lie in an opposite transcriptional orientation on the chromosome.

**Fig. 5:**
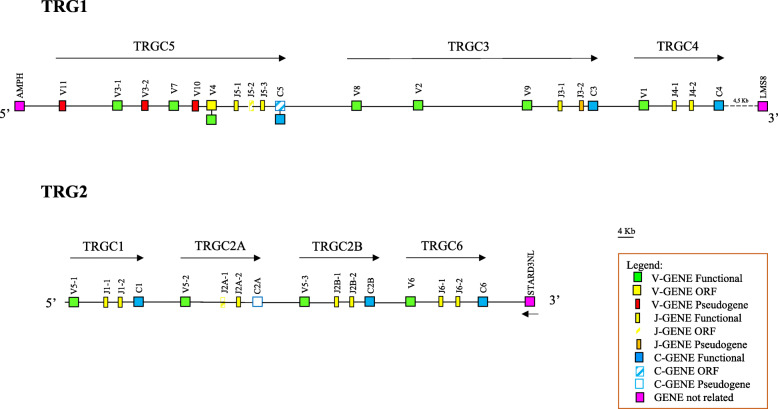
Schematic representation of the genomic organization of the goat TRG1 and TRG2 loci. The diagram shows the position of all the related and unrelated *TRG* genes according to nomenclature. The boxes representing the genes are not to scale. The exons are not shown. The arrows indicate the transcriptional orientation of the genes. The functional *TRGV4* and *TRGC5* genes found in CHIR_2.0 genomic assembly are also indicated (see Results)

The TRG1 locus extends for approximately 170 kb and comprises 10 *TRGV*, 7 *TRGJ* and 3 *TRGC* genes distributed in three V-J-J-C cassettes which we have classified, proceeding from the 5′ to 3′ end of the locus, as TRGC5, TRGC3 and TRGC4 in accordance with the corresponding sheep cassettes (Fig. 5). Six *TRGV* genes are located in the TRGC5 cassette, two in the TRGC3 cassette and one in the TRGC4 cassette. Three *TRGV* (*TRGV11*, *TRGV3–2* and *TRGV10*) and two *TRGJ* (*TRGJ5–2* and *TRGJ3–2*) genes are predicted to be pseudogenes, whereas *TRGC5* appears to be an ORF due to its lack of a stop codon (Additional file 11: Table S5). However, by analysing the *TRG* genes in a different goat genome assembly (CHIR_2.0, GenBank ID: CM001713.2), the predicted functionality of each *TRG1* gene was the same, with the exception of *TRGV4* and *TRGC5*, which were both predicted to be functional in the CHIR_2.0 genome assembly (data not shown). Although we cannot rule out that these differences are due to allelic polymorphisms, it is possible that sequencing errors may account for the disparity. In both assemblies, as in other mammalian TRG loci [23], the *Amphiphysin* (*AMPH*) gene represents the IMGT 5′ borne of the goat TRG1 locus, while, the *U6 small nuclear RNA-associated* (*LSM8*) gene, found and at the 3′ end, may be a candidate gene for the IMGT 3′ borne (Fig. 5).

The TRG2 locus is 110 kb long and contains four V-J-J-C cassettes (Fig. 5), one cassette more than the corresponding sheep locus [26, 41]. We classified the four cassettes, proceeding from the 5′ to 3′ end of the locus as TRGC1, TRGC2A TRGC2B and TRGC6 based on sequence analysis and on homology with the corresponding sheep cassettes. Notably, while the last cassette is clearly homologous to the sheep TRGC6 for the exact correspondence between the genes, less obvious is the correspondence of the first three goat cassettes. A sequence comparison by the Kalign program (at https://www.ebi.ac.uk/), showed that these three cassettes conserve a high level of nucleotide identity (> 96%) as result of recent duplication events, with the highest percentage between the second and the third cassettes (97,32%). These data are also supported by the phylogenetic tree shown in Additional file 12: Figure S4, in which the goat TRGC1, TRGC2A TRGC2B are compared to each other and to the corresponding sheep TRGC1 and TRGC2 cassettes. The tree shows that the goat and sheep sequences form two distinct monophyletic groups in accordance with the phylogeny. Moreover, the goat TRGC2A and TRGC2B cassettes are tightly related and form a paraphyletic group with respect to the TRGC1 cassette in the same species. For this reason, we classified the second and the third cassettes as TRGC2A and TRGC2B, whereas the first cassette is named TRGC1 in accordance with the sheep locus. Unfortunately, the fragmented nature at the 5′ region of the TRG2 locus in CHIR_2.0 prevented us to confirming the presence of the additional cassette in this genomic assembly.

The four cassettes have a typical organization, consisting of one *TRGV*, two *TRGJ* and one *TRGC* genes. All genes are classified as functional, with the exception of the *TRGJ2A-1* ORF and the *TRGC2A* pseudogene (Additional file 11: Table S5). Three out of four *TRGV* genes belong to a same subgroup (TRGV5). Located 15 kb from the last *TRGC* gene is the *Related to steroidogenic acute regulatory protein D3-N-terminal like* (*STARD3NL*) gene. The IMGT 5′ borne is not detectable since no gene has been annotated that could be used as a marker associated with the TRG2 locus. The *cortactin binding protein 2* (*CTTNBP2*) gene was annotated in position 69,617,142..69789671, approximately 495 kb from TRG2.

The classification, position and predicted functionality of all *TRG* genes are reported in Additional file 11: Table S5.

### Classification of the *TRG* genes

Taking into account both TRG loci, a total of 14 germline *TRGV* genes were annotated and assigned to 11 distinct subgroups on the basis of nucleotide identity. All subgroups consist of a single *TRGV* gene, with the exception of the TRGV3 and TRGV5 subgroups, which consist of two and three genes, respectively. The subgroups were classified by comparing the nucleotide sequences of the goat genes and sheep genes (Additional file 13: Figure S5). Therefore, according to the high percentage of nucleotide identity (> 95%), we classified each goat TRGV subgroup in accordance with its corresponding sheep subgroup.

The deduced amino acid sequences of all goat *TRGV* genes, except the *TRGV11* pseudogene, are shown in Additional file 14: Figure S6A with the corresponding sheep genes. The structure of each goat gene is highly similar to that of the corresponding sheep gene with few amino acid variations. As in sheep, the goat TRGV1 and TRGV5 subgroups exhibit leucine 41 instead of tryptophan 41 in FR2-IMGT. Similarly, the hydrophobic amino acid 89 is leucine, phenylalanine or methionine within FR3-IMGT of the corresponding genes.

Additional file 15: Figure S6B reports the nucleotides and deduced amino acid sequences of all *TRGJ* genes identified in the region. All the functional genes are 50 or 60 bp long, except for *TRGJ5–1* and *TRGJ4–1*, which are 53 and 47 bp long, respectively. The functional genes conserve the canonical FG(N or K)XG(A) amino acid motif, whose presence defines the functionality of J genes. The only exceptions are the *TRBJ5–2* and *TRGJ2A-1* genes, which have a different amino acid in the last position of the motif and were therefore classified as ORFs (Additional file 11: Table S5). The *TRGJ3–2* is the only pseudogene, based on the presence of a stop codon. Each *TRGJ* gene is flanked by a 12 RS at the 5′ end and by a donor splice site at the 3′ end. The RS are less conserved in various positions with respect to the consensus sequence, such as the third nucleotide of the “cac” sequence of the heptamer or the poly-A tract of the *TRGJ6–1* nonamer (Additional file 15: Figure S6B). However, a comparison between J-RS of the goat and the sheep functional *TRGJ* genes shows similar nucleotide sequences in heptamers and nonamers (Additional file 17: Figure S7). The third nucleotide of the “cac” sequence shows a similar frequency pattern between goat and sheep J-heptamers. Similarly, the poly-A tract of the nonamers is comparable.

The structure of the goat *TRGC* genes is similar to that of the orthologous sheep genes (Additional file 16: Figure S6C). The first exon (EX1) encodes the C-domain, while the first part of the connecting region is encoded by one (EX2A), two (EX2A and EX2C) or three (EX2A, EX2B and EX2C) exons. As in the sheep gene sequences, the EX2A of all *TRGC* genes (excluding the *TRGC5* gene) contains a TTE(K or G)P(A) P motif in single, duplicate or triplicate form. The only difference between the goat and sheep sequences is the presence of a duplicate motif within the goat *TRGC1* gene where the corresponding sheep gene contains a single motif (Additional file 16: Figure S6C). The remaining portion of the connecting region, the transmembrane region, and the cytoplasmatic region are encoded by EX3.

### Comparison of the goat and sheep TRG loci

We compared the genomic structure of the goat and sheep TRG loci by aligning the whole goat sequences (from the first *TRGV* gene to the last *TRGC* gene located at the 3′ end) with their sheep TRG1 (Additional file 18: Figure S8A) and TRG2 (Additional file 19: Figure S8B) counterparts using the PipMaker program and evaluating their alignment, which is expressed as a dot-plot sequence comparison graph.

The resulting goat/sheep TRG1 locus dot-plot matrix shows an almost perfect main diagonal line, with only a few small interruptions, indicating a high level of identity for which each base matches with itself. The largest break is due to a deletion in the sheep TRGV2-TRGV9 region (blue triangle in Additional file 18: Figure S8A). Moreover, parallel lines of similarity were found in correspondence with each of the J-J-C regions (red boxes), which comprise the enhancer-like (En) elements that were found at the 3′ end of each sheep TRGC gene (green rectangles). Reduced lines of similarity with the J-J-C-En regions are also present between the *TRGV2* and *TRGV9* genes (intermittent red line box in Additional file 18: Figure S8A), likely due to a reminiscence of a complete cassette. Furthermore, the V-cluster of the goat and sheep TRGC5 cassettes show extensive collinearity between them with no evident similarity lines with the TRGV gene sequences of the TRGC3 and TRGC4 cassettes. This is in accordance with the hypothetical evolutionary model for the ovine TRG loci described by Vaccarelli et al. [26] that designates the *V* genes of the sheep TRGC5 cassette as the result of ancestral duplicative events involving only these *V* genes. Conversely, dashes of homology (blue boxes in Additional file 18: Figure S8A) highlight the similarity between the *TRGV* genes belonging to the goat and sheep TRGC3 and TRGC4 cassettes.

Additionally, the comparison of the genomic structure of the goat and sheep TRG2 loci shows an evident similarity confirmed by the length of the uninterrupted lines corresponding to the homologous gene cassettes (Additional file 19: Figure S8B). It is possible to trace along the matrix a continuous main pseudo-diagonal starting from the continuous homology line between the TRGC1 cassette (arrow 1 in Additional file 19: Figure S8B) across the two parallel lines of homology between the goat TRGC2A and TRGC2B and the single sheep TRGC2 cassette (red rectangle in Additional file 19: Figure S8B), to the corresponding homologous line between the TRGC6 cassettes (arrow 3 in Additional file 19: Figure S8B). The only interruptions are due to the missing EX2 in the *TRGC2A* gene with respect to the sheep *TRGC1* and *TRGC2* genes and to a deletion in the sheep TRGJ6-TRGC6 region (violet triangles in Additional file 19: Figure S8B).

Finally, the homology of each cassette in the TRG2 locus extends in correspondence of the sheep enhancer sequences (green rectangles in Additional file 19: Figure S8B).

## Discussion

Goats (*Capra hircus*), one of the most important livestock animals, were domesticated from a single wild ancestor 10,000 years ago [42]. Since then, due to their ability to adapt to various territories, goats have accompanied humans in all activities and have spread all over the world [43]. During domestication, some specialized goat breeds with high performance in many characteristics were generated which now provide important resources for humans. Nevertheless, genetic research on goat traits is lacking, and the limited information we currently have is mostly derived from sheep studies.

With the goal of contributing to our knowledge of goats, we performed a study in *Capra hircus* about the genomic organization of the TRB and TRG loci, which revealed a surprising example of gene family expansion in ruminant species studied so far (i.e., sheep and cattle).

Given the complexity of the TR loci, the recent release of the highly contiguous reference goat genome generated by a combination of methods [44] improving the previous whole genome shotgun assembly [45] was a further stimulus for our analysis of the goat TR loci.

As expected, the general genomic organization of goat TRB is similar to that of the other artiodactyl species [11–14, 16, 17], with three in-tandem D-J-C clusters located downstream of an array of *TRBV* genes and upstream of a single *TRBV* gene, which is positioned in an inverted transcriptional orientation (Fig. 1). Analysis of a publicly available pool of goat cDNAs has shown that the presence of three D-J-C clusters offers to this species, as in the other artiodactyls, a biological advantage from the improved combinatorial and junctional diversity of the CDR3 domain, involved in the antigen binding site, that results from the increased number of the *TRBD* and *TRBJ* genes (Fig. 4). Moreover, expression analysis has reported that, beside the intra-cluster rearrangements, TRBD/TRBJ inter-cluster rearrangements occur during TRB recombination, thus increasing once more the functional diversity of the TR β repertoire.

In contrast to the great diversity in the combinatory region, the three *TRBC* genes are highly similar to each other and to the other *TRBC* genes of artiodactyl species. This conservation may reflect a strong functional constraint linked to the role of the TR constant domain in signal transduction or in interactions with other molecules on the cell surface [46, 47].

The structure of the TRBV region appears, however, to be unique within the goat TRB locus. Ninety-one goat *TRBV* genes, grouped into 27 distinct subgroups, lie in a region of approximately 434 kb. Of these, 182 kb are occupied by a massive expansion of the TRBV5 and TRBV6 gene subgroups, with 30 and 29 members, respectively. The 6 gene members of the TRBV21 subgroup represent the other multimember subgroup. As shown in the locus map (Fig. 1), the *TRBV5* and *TRBV6* genes are alternated and intermingled within the genomic region, indicating that shared duplicative events contributed to their extensive expansion, unlike the *TRBV21* genes that are clustered together due to tandem duplications occurring from a single ancestral gene. Dot-plot analyses of the duplicate TRBV region reinforce the conclusion that the TRBV5 and TRBV6 genomic organization arose through a series of complex tandem duplication events, which rarely involved either the *TRBV5* or the *TRBV6* gene, but, more frequently, involved genes from both subgroups generating duplication units of different sizes (Additional file 5-6: Figure S2A-B).

The same extensive gene duplications occur in the sheep and bovine TRB loci [11, 13]. In sheep, 94 *TRBV* genes have been identified and grouped into 26 subgroups. Among these genes are 33 *TRBV5* and 30 *TRBV6* genes, which are intermingled as observed in the sheep locus. Also in this species, six clustered genes form the TRBV21 subgroup. In this regard, our phylogenetic study (Fig. 3) demonstrates that the gene duplications occurred during the shared evolutionary history of sheep and goat. In fact, most of the sheep *TRBV5*, *TRBV6* and *TRBV21* genes correspond to the orthologous goat genes, indicating that the duplication events occurred in a common ancestor of the two species.

In cattle, although the TRB sequence in the third bovine assembly seems incomplete [13], 134 *TRBV* genes, distributed among 24 subgroups have been found. In this case, the major germline repertoire is also attributable to the expansion of two TRBV subgroups whose genes are alternated at the 5′ of the V-cluster. One subgroup is the TRBV6, as in goat and sheep, which consists of 40 members; the other subgroup, with 35 members, is classified as TRBV9 and likely corresponds to the goat TRBV5. As a matter of a fact, the same authors mention that the identity between the nucleotide sequences of the *TRBV9* and *TRBV5* genes is often > 75%. In addition, dot-plot analyses of the bovine TRB locus (Fig. 3 in [13]) show the same duplication scheme observed in the goat TRBV5 and TRBV6 genomic region (Additional file 5-6: Figure S2A-B). Furthermore, the bovine TRBV21 subgroup contain 16 members which, unlike in sheep and goat, appears to have been generated by duplications that also involved the bovine *TRBV18*, *TRBV19* and *TRBV20* genes [13].

If ancient gene duplications within the TRB locus led to the generation of the different TRBV subgroups shared among mammals [48], in ruminants the framework of the TRBV germline repertoire evolved with a more recent expansion of two main TRBV subgroups rather than with the emergence of diverse TRBV subgroups. Overall, this extensive gene expansion resulted in ruminant species (goat, sheep and cattle) possessing a germline TRBV repertoire with the highest number of genes among all the mammalian species studied so far [7, 11], including other artiodactyl species such as pigs and camels [14–17].

For comparison, it is interesting to note that TRBV5, TRBV6 and TRBV21 are also multimember subgroups in rabbits [8, 11], which possess 17 *TRBV5*, 14 *TRBV6* and 7 *TRBV21* genes. Humans and rhesus monkey possess major expansion in TRBV5 and the TRBV6 [11], though the gene number of each subgroup is never as high as in ruminants. The human TRB locus contains 8 *TRBV5* and 9 *TRBV6* genes, whereas 10 *TRBV5* and 8 *TRBV6* genes are present in rhesus monkey.

However, the ruminant functional TRB repertoire is strongly conditioned by the proportion of no- functional germline *TRBV5* and *TRBV6* genes. In fact, the percentage of no-functional goat *TRBV5* genes is 40% (12/30), while 62% (18/29) of the TRBV6 subgroup genes are no-functional. The percentage of no-functional genes for the two subgroups is similarly high in sheep and cattle: in sheep, 57.5 and 66.6% of *TRBV5* and *TRBV6* genes, respectively, are no-functional, whereas in cattle, 50 and 34.2% of *TRBV6* and *TRBV9* genes are no-functional. Therefore, it appears that the gene expansion of these subgroups might be related not to specific functional needs but rather to the scheme of gene duplications. In contrast, it has been reported [19, 22] that the sheep and cattle TRD repertoire is clearly determined by the high percentage of functional germline genes belonging to the multimember TRDV1 subgroup, where the percentage of non-functional genes is very low (22% (6/27) in sheep and 14.2% (8/56) in cattle).

The organization of the *TRG* genes into two distinct and separate genomic regions was already known in sheep [26, 27] and cattle [24, 25]. However, in both species, the genomic structures of the two paralogous TRG loci was archived by analysis of BAC clones, and the structural relationship between them had not yet been determined. The deduced genomic organization of the TRG loci from the goat genomic assembly allowed us to establish the precise chromosomal position of the two loci, their distance and their reciprocal transcriptional orientation. Moreover, in all mammalian species with a single TRG locus, the *AMPH* and *STARD3NL* genes represent the IMGT 5′ and IMGT 3′ borne, respectively, since they are located upstream of the first and downstream of the last *TRG* gene (IMGT®, http://www.imgt.org). In goat, and likely other ruminants, the synteny has been broken as a consequence of the evolutionary TRG split, with the *AMPH* located at the 5′ end of the TRG1 locus and the *STARD3NL* gene at the 3′ end of the TRG2 locus (Fig. 5). Taking into account that an intrachromosomal transposition seems to have moved the *TRG2* genes to the current 4q15–22 position [27, 41], this implies that the split also involved the *STARD3NL* gene. Therefore, in goat and likely all ruminant species, two more gene boundaries should be defined: the IMGT 3′ borne of the TRG1 and the IMGT 5′ borne of the TRG2 locus. We propose the *LSM8* gene located 4.5 kb from the *TRGC4* gene as the 3′ borne of the TRG1 locus, whereas no gene was found in the vicinity of the *TRGV5–1* gene to be proposed as the 5′ borne of the TRG2 locus.

The molecular characterization of the goat TRG loci showed that the TRG1 locus is very similar to the corresponding sheep locus in terms of gene content and genomic organization (Fig. 5 and Additional file 14-15: Figure S6A-B). The comparison between the goat and sheep TRG1 sequences (Additional file 18: Figure S8A), however, revealed, between *TRGV2* and *TRGV9* genes, homology traces with the J-C regions, likely due to an additional cassette, which is still present in the same position in the bovine TRG1 locus (TRGC7 cassette in https://www.imgt.org/IMGTrepertoire/).

An additional functional V-J-J-C cassette is, however, present in the goat TRG2 locus compared to that of sheep and cattle, as result of a recent duplication event involved the ancestral TRGC2 cassette giving rise to TRGC2A and TRGC2B. As a matter of a fact, the two TRGC2 cassettes show the highest nucleotide similarity between them (> 97%) even if the presence of a complete deletion of EX2 in the *TRGC2A* gene probably makes this cassette not functional.

In line with the evolutionary scenario proposed by Vaccarelli et al., [26] for the formation of the sheep TRG loci, we hypothesize that similar reiterated in-tandem duplications of V-J-J-C units may also have generated the goat TRG loci. Briefly, after the duplication of a minimum ancestral cassette consisting of one *V*, three *J* and one *C* gene, the ancestral TRG locus consisted of two cassettes, that were likely the forerunners of the TRGC5 and TRGC6 cassettes and were bordered by the *AMPH* and *STARD3NL* genes at their 5′ and 3′ ends, respectively. Subsequently, the TRGC5 cassette formed the TRGCC3 cassette, which in turn duplicated to generate the TRGC4 cassette. A duplication of the TRGC4 cassette produced the TRGC2 cassette, which in turn generated the TRGC1 cassette. At this point, in the goat TRG2 locus, a further duplicative event involving the TRGC2 cassette may have generated the fourth cassette. However, given the high identity between the TRGC2A, TRGC2B and TRGC1 cassettes, it is also possible that the additional cassette resulted from an unequal crossing-over event between the ancestral TRGC1 and TRGC2 cassette, and the reworked *TRGC2A* pseudogene may represent the outcome of this event. Unequal crossing-over events have previously been evoked in artiodactyls as the origin of the third TRBD-J-C cluster [12, 13, 49].

## Conclusions

The annotation of the goat TRB and TRG loci in ARS1 is an important achievement. Overall, these findings show that in goats, as well as in sheep and cattle, the TRB and TRG loci are characterized by extensive gene expansion events that provide the germline TRB and TRG repertoire, suggesting that a strong evolutionary pressure has driven the enlargement of the *TR* genes, and thus produced greater potential TR diversity in the ruminant lineage. However, further studies will be required to define the full extent of these expansions and to understand their evolutionary basis taking into account the high “death rate” in the main TRBV subgroups that seems to affect the germline repertoire.

Furthermore, it could be interesting to evaluate from an evolutionary point of view whether the expansion of the *TRB* and *TRG* genes results a trait of all ruminant species or concerns only the Bovidae family, i.e., goat, sheep and cattle.

## Methods

### Genome analyses

To determine the TRB and the TRG locus location, the latest version of the goat ARS1 genome sequence was searched using the BLAST algorithm.

For TRB, a sequence of 557,687 bp was retrieved directly from the reference sequence NC_030811.1 (*Capra hircus* chromosome 4 genomic scaffold) available at NCBI from 14,442,487 to 17,000,174 positions. Particularly, the analysed region comprises the *monooxygenase, dopamine-beta-hydroxylase-like 2* (*MOXD2*) and *ephrin type-B receptor 6* (*EPHB6*) genes, flanking respectively, the 5′ and 3′ ends of the TRB locus.

For TRG, two sequences, respectively of 169,887 bp (TRG1) and 143,000 bp (TRG2), were retrieved directly from the same NC_030811.1 reference sequence available at NCBI, from 37,490,000 to 37,659,887 (complement, TRG1) and from 70,113,000 to 70,256,000 (TRG2) positions.

The available sheep TRB and TRG genomic sequences [11, 12, 41, 50] were used against the *Capra hircus* genome sequences to identify, based on homology by the BLAST program, the corresponding genomic *V*, *D*, *J* and *C* genes. Moreover, the homology-based method was used, aligning the goat retrieved sequence against itself with the PipMaker program [51]. The beginning and end of each coding exon were identified with accuracy by the presence of splice sites or flanking recombination signal (RS) sequences of the *V*, *D* and *J* genes.

Locations of the *TRB* and *TRG* genes are provided in Additional file 1: Table S1 and Additional file 11: Table S5. The sequence comparison has also allowed the identification and characterization of the goat protease genes (*TRY*) within the TRB locus. The locations of the *TRY* genes are provided in Additional file 2: Table S2.

Moreover, computational analysis of the goat TRB and TRG loci was conducted using the RepeatMasker for the identification of genome-wide repeats and low complexity regions (Smit, A.F.A. Hubley, R. Green, P. RepeatMasker open-4.0. at http://www.repeatmasker.org). The PipMaker program was also used for the genomic comparative analyses for the alignment of the goat TRB sequence with itself and of the goat TRG loci with the sheep genomic corresponding TRG1 (GenBank I. D.: DQ992075) and TRG2 (GenBank I. D.: DQ992074) sequences.

### Classification of the goat TR genes

Considering the percentage of nucleotide identity of the genes with respect to human and the other artiodactyl species and based on the genomic position within the locus, each goat *TRB* as well as *TRG* gene was classified, and the nomenclature was established according to IMGT at http://www.imgt.org/IMGTScientificChart/SequenceDescription/IMGTfunctionality.html [52] (see Additional file 1: Table S1 and Additional file 11: Table S5). The functionality of the *V*, *D*, *J* and *C* genes was predicted through the manual alignment of sequences adopting the following parameters: (a) identification of the leader sequence at the 5′ of the *V* genes; (b) determination of proper RS sequences located at 3′ of the *V* (V-RS), 5′ and 3′ ends of the *D* (5’D-RS and 3’D-RS) and 5′ of the *J* (J-RS), respectively; (c) determination of conserved acceptor and donor splicing sites; (d) estimation of the expected length of the coding regions; (e) absence of frameshifts and stop codons in the coding regions of the genes. Conversely, a germline gene is qualified as ORF (Open Reading Frame) if the coding region has an open reading frame, but alterations have described in the splicing sites and/or RS sequences, and/or in changes of conserved amino acids. Finally a germline gene is qualified as pseudogene (P) if its coding region has stop codon(s) and /or frameshift mutation(s).

The *TRBV* and *TRGV* genes were respectively assigned to 27 and 11 different subgroups, based on the percentage of nucleotide identity by using Clustal Omega alignment tool, which is available at EMBL-EBI website (http://www.ebi.ac.uk/), adopting the criterion that sequences with a nucleotide identity of more than 75% in the V-REGION belong to the same subgroup [32].

The *TRBD*, *TRBJ* and *TRBC* genes were annotated, according to the similarity with the other artiodactyl species [11–17]. Each *TRBJ1*, *TRBJ2* and *TRBJ3* gene was designed by a hyphen and a number corresponding to their position in the cluster except for the *TRBJ3–6* that precedes the *TRBJ3–5* gene as in the sheep locus [11]. They were all predicted to be functional except for the *TRBJ1–3* and *TRBJ3–6* (Additional file 1: Table S1).

The *TRGJ* genes were named by a number in accordance to name of the belonging TRGC cassette, followed by a hyphen and a number corresponding to their position within the cassette.

### Phylogenetic analyses

The *TRBV* genes used for the phylogenetic analysis were retrieved from the following sequences deposited in the GEDI (for GenBank/ENA/DDBJ/IMGT/LIGM-DB) databases: NC_040255 (sheep TRB locus contig as annotated in IMGT000042, IMGT database, [11]) NW_011591622, NW_011593440, NW_011591151, NW_011620189, NW_011616084, NW_011607149, NW_011601111 and LT837971 (dromedary TRB locus contig as characterized by [14, 15]; NC_010460 (pig TRB locus contig as characterized by [16]); and NC_030811.1 (this work) (goat TRB locus contig). We combined the nucleotide sequences of the V-REGION of the goat *TRBV* genes with the corresponding gene sequences of sheep, pig and dromedary.

Multiple alignments of the gene sequences under analysis were carried out with the MUSCLE program [53]. The evolutionary analyses were conducted in MEGA 7 [35]. We used the neighbour-joining (NJ) method to reconstruct the phylogenetic tree [34]. The evolutionary distances were computed using the p-distance method [37] and are in the units of the number of base differences per site.

### cDNA analysis of public collection

Goat β chain cDNAs were retrieved from IMGT public database (https://www.imgt.org/ligmdb/) and were aligned with the germline TRB sequences determined in this study. A total of 27 clones derived from peripheral blood, were analysed in detail (Additional file 10: Table S4 and Fig. 4). The expressed *TRBV* genes have been assigned to the corresponding germline *TRBV* genes and, on the basis of the percentage of nucleotide identity, classified as allele*01 if the sequence perfectly matched the germline sequence and as allele*02 or *03 when the percentage ranging from 97 to 99%.

The CDR3 size within the cDNA clones was calculated by the number of amino acids between the amino acid after the conserved 2nd cysteine in the *V* gene (pos.104), and the amino acid before the phenylalanina of the FGXG motif in the *J* gene (http://www.imgt.org, [52]).

## Supplementary information

**Additional file 1: Table S1.** Description of the *TRB* genes in the *Capra hircus* chromosome 4 genome assembly (NCBI Reference Sequence CM_004565.1). The position of all genes and their classification and functionality are reported.

**Additional file 2: Table S2.** Description of the unrelated *TRB* genes in the *Capra hircus* chromosome 4 genome assembly (NCBI Reference Sequence CM_004565.1). The position of all genes and their classification and functionality are reported.

**Additional file 3: Table S3.** Description of the *Caphir TRBV* pseudogenes and ORF.

**Additional file 4: Figure S1.** Description of the goat *TRBV* genes. The IMGT Protein display of the goat *TRBV* genes. The deduced amino acid sequences of the goat *TRBV* genes were manually aligned according to IMGT unique numbering for the V-REGION [33] to maximize homology. Only functional genes, ORF and in-frame pseudogenes are shown. All sequences exhibit the typical framework regions (FR) and complementarity determining regions (CDR) and the four amino acids (depicted and indicated in bold): cysteine 23 (1st-CYS) in FR1-IMGT, tryptophan 41 (CONSERVED-TRP) in FR2-IMGT, hydrophobic (here L and M) 89, and cysteine 104 (2nd-CYS) in FR3-IMGT, with the exception of *TRBV6–20* and *TRBV6–21* that lack the CONSERVED-TRP and the 2nd-CYS, respectively. Conversely, CDR-IMGT vary in amino acid composition and length. The description of the strands and loops and of the FR-IMGT and CDR-IMGT is according to the IMGT unique numbering for V-REGION [33]. The amino acid length of the CDR-IMGT AA is also indicated in square brackets. (B-D), nucleotide and deduced amino acid sequences of the *TRBD*, *TRB*J and *TRDC* genes. The consensus sequence of the heptamer and nonamer is provided at the top of the figure and is underlined. The numbering adopted for the gene classification is reported on the left of each gene.

**Additional file 5: Figure S2. (A)** Dot-plot of the goat TRBV cluster sequence against itself. Description: With the exception of the main diagonal line for the match of each base with itself, dots and diagonal lines indicate internal homology units in the sequence. The red boxes show the TRBV regions underwent to duplication events.

**Additional file 6: Figure S2. (B)** Dot-plot of the goat TRBV cluster sequence against itself. Description: Enlargement of the wider red box in (A) showing the pattern of parallel lines due to the tandem duplicative events of the *TRBV5* and *TRBV6* genes. The arrows indicate long homology lines determined by a repetitive TRBV5-TRBV6 base unit.

**Additional file 7: Figure S3. (A)** Description of the goat *TRBD* genes. The inferred amino acid sequence of the *TRBD* genes in the three coding frames are reported.

**Additional file 8: Figure S3. (B)** Description of the goat *TRBJ* genes. The donor splice site for each *TRBJ* is shown. The canonical FGXG amino acid motifs are underlined. The *TRBJ1–3* and *TRBJ2–7* ORF and *TRB3–6* pseudogene are indicated in italics.

**Additional file 9: Figure S3. (C)** Description of the goat *TRBC* genes. The IMGT Protein display of the goat *TRBC* gene compared with the human, sheep, pig and dromedary Cβ proteins. The descriptions of the strands and loops were collected according to the IMGT unique numbering for the C-DOMAIN [54].

**Additional file 10: Table S4.** Correspondence between the germline and the expressed *TRBV* genes. Description: The nucleotide identity, the Accession number and the IMGT name of each corresponding cDNA clone are reported.

**Additional file 11: Table S5.** Description of the related and unrelated *TRG* genes in the *Capra hircus* chromosome 4 genome assembly (NCBI Reference Sequence NC_030811.1). The position of all genes and their classification and functionality are reported.

**Additional file 12: Figure S4.** The NJ tree inferred from the genomic sequences of the goat TRGC1, TRGC2A and TRGC2B cassettes, together with the sheep TRGC1 and TRGC2 cassette sequences. Description: The evolutionary analysis was conducted in MEGA7 [35]. The optimal tree with the sum of branch length = 0.08464556 is shown. The percentage of replicate trees in which the associated taxa clustered together in the bootstrap test (100 replicates) is shown next to the branches [36]. The tree is drawn to scale with branch lengths in the same units as those of the evolutionary distances used to infer phylogenetic trees. The evolutionary distances were computed using the p-distance method [37] and are in the units of the number of base differences per site. The analysis involved 5 nucleotide sequences. All positions containing gaps and missing data were eliminated. There were a total of 17,718 positions in the final dataset.

**Additional file 13: Figure S5.** Percent Identity Matrix created by CLUSTAL. Description: Output file obtained by the multiple alignment of the goat and sheep TRGV gene sequences.

**Additional file 14: Figure S6. (A)** Description of the goat *TRGV* genes. Description: The IMGT Protein display of the goat *TRGV* genes compared with the sheep orthologous genes. The deduced amino acid sequences of the *TRGV* genes were manually aligned according to IMGT unique numbering for the V-REGION [33] to maximize homology. Only functional genes, ORF and in-frame pseudogenes are shown. All sequences exhibit the typical framework regions (FR) and complementarity determining regions (CDR) and the four amino acids (indicated in bold): cysteine 23 (1st-CYS) in FR1-IMGT, tryptophan 41 (CONSERVED-TRP) in FR2-IMGT, with the exception of TRGV1 and TRGV5 subgroups, hydrophobic (here L, F and M) 89, and cysteine 104 (2nd-CYS) in FR3-IMGT. Conversely, CDR-IMGT vary in amino acid composition and length. The description of the strands and loops and of the FR-IMGT and CDR-IMGT is according to the IMGT unique numbering for V-REGION [33].

**Additional file 15: Figure S6. (B)** Description of the goat *TRGJ* genes. Description: Nucleotide and deduced amino acid sequences of the *TRBJ* genes**.** The consensus sequence of the heptamer and nonamer is provided at the top of the figure and is underlined. The numbering adopted for the gene classification is reported on the left of each gene. The donor splice site for each *TRBJ* is shown. The canonical FGXG amino acid motifs are underlined. The *TRGJ3–2* pseudogene and the *TRGJ5–2* and *TRGJ7–1* ORF are indicated in italics.

**Additional file 16: Figure S6. (C)** Description of the goat *TRGC* genes. Description: The IMGT Protein display of the goat TRGC gene compared with the sheep Cγ proteins. The descriptions of the strands and loops were collected according to the IMGT unique numbering for the C-DOMAIN [54].

**Additional file 17: Figure S7. J-RS sequence logos.** Description: Recombination signal sequence logos of the heptamers and nonamers of the goat and sheep *TRGJ* genes generated using Weblogo [55] The height of letters indicates the relative frequency of each nucleotide at that position.

**Additional file 18: Figure S8. (A)** Dotplot matrix of goat/sheep TRG1 genomic comparison. Description: The transcriptional orientation of each gene is indicated by arrows and arrow-heads. The triangle indicates a gap (insertions or deletions) between the homology unit. Coloured boxes enclose J-C blocks (red), *TRGV* genes (blue) and enhancer-like sequences (green).

**Additional file 19: Figure S8. (B)** Dotplot matrix of goat/sheep TRG2 genomic comparison. Description: The transcriptional orientation of each gene is indicated by arrows and arrow-heads. The triangle indicates a gap (insertions or deletions) between the homology unit. The regions of more extensive homology are numbered. The parallel lines representing the duplication of TRGC2A and TRGC2B cassettes are boxed (red). Green rectangle enclose the position of the enhancer-like sequences in the sheep locus.

## Data Availability

Not applicable.
