# Supplementary material for: The expansion of the TRB and TRG genes in domestic goats (Capra hircus) is characteristic of the ruminant species
Source: BMC Genomics. 2020 Sep 11;21:623. doi: 10.1186/s12864-020-07022-x (PMC7488459; doi:10.1186/s12864-020-07022-x)
Supplement: Supplementary file 1 — Additional file 1: Table S1. Description of the TRB genes in the Capra hircus chromosome 4 genome assembly (NCBI Reference Sequence CM_004565.1). The position of all genes and their classification and functionality are reported. [file 12864_2020_7022_MOESM1_ESM.pdf]

Table S1. Description of the *TRB* genes in the *Capra hircus* chromosome 4 genome assembly (NCBI Reference Sequence CM_004565.1). The position of all genes and their classification and functionality are reported.

**Gene classification**	**Functionality**	**Position**[a] (complement)
TRBV1	F	14984015-14984322
TRBV3	F[b]	14907378-14907838
TRBV4	F	14903033-14903488
TRBV2	F	14898809-14899260
TRBV5-1	P	14891298-14891689
TRBV5-2	F	14884857-14885334
TRBV6-1	F	14880001-14880431
TRBV5-3	F	14876102-14876576
TRBV6-2	P	14872197-14872625
TRBV5-4	P	14869595-14870070
TRBV5-5	F	14866561-14867033
TRBV6-3	P	14864812-14865241
TRBV5-6	F	14862535-14863010
TRBV5-7	P	14858129-14858600
TRBV6-4	P	14855092-14855522
TRBV5-8	F	14852745-14853220
TRBV6-5	F	14850941-14851370
TRBV6-6	F	14847814-14848244
TRBV5-9	F	14844870-14845326
TRBV6-7	P	14840298-14840725
TRBV5-10	P	14837991-14838462
TRBV6-8	P	14836310-14836739
TRBV5-11	P	14833988-14834459
TRBV6-9	P	14831500-14832129
TRBV5-12	F	14829163-14829639
TRBV6-10	P	14827490-14827937
TRBV5-13	P	14825745-14826215
TRBV6-11	F	14823870-14824298
TRBV5-14	F	14821654-14822111
TRBV6-12	P	14817035-14817458
TRBV5-15	P	14814687-14815165
TRBV6-13	P	14812871-14813299
TRBV5-16	F	14810546-14811023
TRBV6-14	P	14808147-14808573
TRBV5-17	F	14805577-14806053
TRBV6-15	P	14803917-14804352
TRBV5-18	F	14801694-14802151
TRBV6-16	F	14799888-14800318
TRBV6-17	ORF	14796555-14796986
TRBV5-19	F	14793458-14793932
TRBV6-18	F	14791344-14791772
TRBV6-19	F	14788164-14788593
TRBV5-20	P	14786407-14786878
TRBV6-20	ORF	14784505-14784933
TRBV5-21	F	14782273-14782730
TRBV6-21	ORF	14777737-14778160
TRBV5-22	F	14775382-14775860
TRBV6-22	P	14773555-14773985

TRBV5-23	P	14771163-14771727
TRBV6-23	ORF	14768372-14768802
TRBV6-24	P	14765013-14765442
TRBV5-24	F	14762789-14763246
TRBV6-25	P	14754413-14754840
TRBV5-25	P	14752079-14752554
TRBV6-26	F	14750394-14750821
TRBV5-26	P	14748092-14748563
TRBV6-27	F	14745817-14746247
TRBV5-27	F	14743611-14744069
TRBV5-28	P	14736819-14737282
TRBV6-28	F	14734992-14735419
TRBV5-29	F	14732653-14733130
TRBV6-29	F	14729739-14730168
TRBV7-1	F	14726886-14727332
TRBV8	P	14722863-14723353
TRBV5-30	F	14714773-14715240
TRBV7-2	F	14709813-14710296
TRBV9	P	14704699-14704981
TRBV10	P	14698951-14699383
TRBV11	P	14694487-14694943
TRBV12-1	F	14687500-14687943
TRBV12-2	F	14672039-14672481
TRBV14	P	14669550-14669978
TRBV15	F	14667225-14667689
TRBV16	F	14662154-14662607
TRBV18	P	14648592-14649224
TRBV19	F	14646735-14647211
TRBV20	F	14641339-14642152
TRBV21-1	F	14634677-14635133
TRBV21-2	F	14629352-14629795
TRBV21-3	F	14620320-14620770
TRBV21-4	F	14615849-14616310
TRBV21-5	F	14611937-14612387
TRBV21-6	F	14605623-14606081
TRBV22	F	14601178-14601645
TRBV24	F	14595644-14596117
TRBV25	F	14589280-14589750
TRBV26	F	14587349-14587828
TRBV27	P	14575967-14576435
TRBV28	F	14569871-14570543
TRBV29	F	14562392-14562986
TRBD1	F	14543107-14543120
TRBJ1-1	F	14542403-14542448
TRBJ1-2	F	14542272-14542315
TRBJ1-3	ORF	14541977-14542024
TRBJ1-4	F	14541399-14541447
TRBJ1-5	F	14541123-14541172
TRBJ1-6	F	14540328-14540380
TRBC1	F	14535962-14537639
TRBD3	F	14533308-14533321
TRBJ3-1	F	14532614-14532663
TRBJ3-2	F	14532463-14532513
TRBJ3-3	F	14532247-14532295

TRBJ3-4	F	14532130-14532177
TRBJ3-6	P	14532042-14532094
TRBJ3-5	F	14531825-14531871
TRBC3	F	14526671-14528093
TRBD2	F	14523578-14523593
TRBJ2-1	F	14522522-14522571
TRBJ2-2	F	14522325-14522375
TRBJ2-3	F	14522107-14522155
TRBJ2-4	F	14521960-14522008
TRBJ2-5	F	14521843-14521888
TRBJ2-6	F	14521740-14521792
TRBJ2-7	ORF/F[c]	14521509-14521554
TRBC2	F	14517246-14518875
TRBV30	F	14503203-14503862

[a] L-PART1/ V-exon for TRBV genes

[b] STOP-CODON at position 108 (last 3' codon of germline CDR3-IMGT) may disappear during rearrangements

[c] J-GENE functional is referred to TRBJ2-7 detected within cDNAs
