# Supplementary material for: The expansion of the TRB and TRG genes in domestic goats (Capra hircus) is characteristic of the ruminant species
Source: BMC Genomics. 2020 Sep 11;21:623. doi: 10.1186/s12864-020-07022-x (PMC7488459; doi:10.1186/s12864-020-07022-x)
Supplement: Supplementary file 2 — Additional file 2: Table S2. Description of the unrelated TRB genes in the Capra hircus chromosome 4 genome assembly (NCBI Reference Sequence CM_004565.1). The position of all genes and their classification and functionality are reported. [file 12864_2020_7022_MOESM2_ESM.pdf]

Table S2. Description of the unrelated TRB genes in the *Capra hircus* chromosome 4 genome assembly (NCBI Reference Sequence CM_004565.1). The position of all genes and their classification and functionality are reported.

**Gene classification**	**Functionality**	**Position** (complement)
MOXD2	F	14992271-15000174
TRY1	F	14973346-14979051
TRY2	F	14962597-14969667
TRY3	F	14951833-14955674
TRY4	F	14927506-14931123
TRY5	F	114553420-4549667
EPHB6	F	14442487-14449079
