# Supplementary material for: The expansion of the TRB and TRG genes in domestic goats (Capra hircus) is characteristic of the ruminant species
Source: BMC Genomics. 2020 Sep 11;21:623. doi: 10.1186/s12864-020-07022-x (PMC7488459; doi:10.1186/s12864-020-07022-x)
Supplement: Supplementary file 3 — Additional file 3: Table S3. Description of the Caphir TRBV pseudogenes and ORF. [file 12864_2020_7022_MOESM3_ESM.pdf]

Table S3. Description of the *Caphir TRBV* pseudogenes and ORF.

TRBV genes	no INIT-CODON	Defective leader	Frameshift in V-REGION	Stop codon	Defective splice site	Defective RS	no CONSERVED-CYS or TRP
TRBV5-1			•	•		•	
TRBV5-4				•			
TRBV5-7			•				
TRBV5-10			•				
TRBV5-11				•			
TRBV5-13			•				
TRBV5-15				•			
TRBV5-20			•				
TRBV5-23			•				
TRBV5-25			•			•	
TRBV5-26			•				
TRBV5-28				•			
TRBV6-2	•			•			
TRBV6-3				•			
TRBV6-4				•		•	
TRBV6-7				•			
TRBV6-8			•				
TRBV6-9			•	•			
TRBV6-10			•				
TRBV6-12			•			•	
TRBV6-13				•			
TRBV6-14			•	•		•	
TRBV6-15			•			•	
TRBV6-17					•	•	
TRBV6-20							•
TRBV6-21							•
TRBV6-22				•			
TRBV6-23						•	
TRBV6-24				•			
TRBV6-25				•			
TRBV8		•	•				
TRBV9		•	•				
TRBV10			•				
TRBV11			•	•			
TRBV14	•						
TRBV18	•	•	•	•		•	
TRBV27	•						
