# Supplementary material for: The expansion of the TRB and TRG genes in domestic goats (Capra hircus) is characteristic of the ruminant species
Source: BMC Genomics. 2020 Sep 11;21:623. doi: 10.1186/s12864-020-07022-x (PMC7488459; doi:10.1186/s12864-020-07022-x)
Supplement: Supplementary file 4 — Additional file 4: Figure S1. Description of the goat TRBV genes. The IMGT Protein display of the goat TRBV genes. The deduced amino acid sequences of the goat TRBV genes were manually aligned according to IMGT unique numbering for the V-REGION [33] to maximize homology. Only functional genes, ORF and in-frame pseudogenes are shown. All sequences exhibit the typical framework regions (FR) and complementarity determining regions (CDR) and the four amino acids (depicted and indicated in bold): cysteine 23 (1st-CYS) in FR1-IMGT, tryptophan 41 (CONSERVED-TRP) in FR2-IMGT, hydrophobic (here L and M) 89, and cysteine 104 (2nd-CYS) in FR3-IMGT, with the exception of TRBV6–20 and TRBV6–21 that lack the CONSERVED-TRP and the 2nd-CYS, respectively. Conversely, CDR-IMGT vary in amino acid composition and length. The description of the strands and loops and of the FR-IMGT and CDR-IMGT is according to the IMGT unique numbering for V-REGION [33]. The amino acid length of the CDR-IMGT AA is also indicated in square brackets. (B-D), nucleotide and deduced amino acid sequences of the TRBD, TRBJ and TRDC genes. The consensus sequence of the heptamer and nonamer is provided at the top of the figure and is underlined. The numbering adopted for the gene classification is reported on the left of each gene. [file 12864_2020_7022_MOESM4_ESM.pdf]

```
                                 L-PART1+L-PART2          FR1-IMGT             CDR1-IMGT          FR2-IMGT        CDR2-IMGT                   FR3-IMGT                         CDR3-IMGT
                                                                (1-26)              (27-38)              (39-55)          (56-65)                        (66-104)                         (105-117)
                                                         A                  B          BC                C          C'          C'C"          C"           D             E              F             FG
                                                      (1-15)             (16-26)    (27-38)         (39-46)     (47-55)     (56-65)      (66-74)      (75-84)       (85-96)       (97-104)     (105-117)
Gene Functionality                                1        10   15  16     23  26 27          38 3941   46 47      55 56       65 66      74 75    80 84 85  89      96 97    104 105
                                                  |........|....|   |......|..|  |..........|  |.|....|   |.......|  |........|  |.......|  |....|...|  |...|.......|  |......|  |.....
TRBV1     F          MALFPVLWGWVLLAAVSTDAA TSLVEQKPRWVLVPH GQAETLRCILK DSQ......YPW MSWYQQDL RGQLQVLAS LRS....TGD EEVINLP.G ANYRATRVSE SELSLHVANVTQ GRTLFCTC SK.... [6.6.2]
TRBV2     F           MDSRLFCWAILCLLGVRHL VPGVSQSPRHYITEM GWDVTLRCDPM PGH.......LD LYWYRQTL GKGMEFLMS IYN....KEP SEKAHFMED CFSAEMP.DG AYLILKMQPAQL GDLAMFLC ASSL.. [5.6.4]
TRBV3     F           MQSGLLCCVALGLLGVGTW GTGVFQTPKYLLTQM GTKTSLECEQK LNH.......DN MYWYKQDS KKLLKAMFH YNN....KEL VENETVP.S RFSPESP.DK AHLNLHIDSLEP GDSAVYFC ASS*.. [5.6.3]
TRBV4     F          MGFRQLCCVVLCLLGIAVSA DPGITQIPKYLVMGM TDKKSLKCEQH LGH.......NA MYWYKQSA QKPPELMFI HSY....MKL SGNESVP.S RFWPECP.DS SQCRLDLSAPKP QDSAVYLC ASSR.. [5.6.4]
TRBV5-2   F           MGSRLLCCVTLCLLGAGLV DSEVTQTPKYLIKSR KEQATLGCSPE SGH.......RS VNWYQQAL GQGPRFLVQ YYR....GQV SGEENMP.D RFSAKQF.SD FRSELNLSSLEL TDSAVYLC ASSQ.. [5.6.4]
TRBV5-3   F           MGSRLLCCVTLCLLGAGLV DSEVTQTPKYLIKSR KQQATLRCSPE SGH.......FS VYWYQQVL GQGPQFLVQ YYN....QKV SGEAHLP.D RFSGKQF.SD SRSELNLSSLEL MDSAMYLC ASSQ.. [5.6.4]
TRBV5-4   P           MGSRLLCCVTVCLLGAGLV DSEVTQTPKYLIKSR QQQVTLSCSPE SAH.......HS VYWYQQAL GQGP*FLVQ YYN....REM IQKGKIS.D RFSGKQF.SD FHSELNLTSLEL TDSAVYLC ASSQ.. [5.6.4]
TRBV5-5   F           MGSRLLCCLTVCLLGAGLV DSEVTQTPKFLTKSR KQQVTLRCSPE SGH.......RS VYWFQQAL GQGPQFLVQ YFD....GKV YQRGNMS.D RFSGQQ.... SGSELNLTSLEL MDSALYLC ASSQ.. [5.6.4]
TRBV5-6   F           MGSRLLCCVTLCLLGAGLV YSGVTQTPKYLIKSR KQQGILRCSPE SGH.......LY VSWYQQAL GQGPQFLVQ YYD....GKV RTKGNMP.D RFSGEQF.SD SHSQMNLSSLKL SDSAVYLC ASSQ.. [5.6.4]
TRBV5-8   F           MGSRLLCCVTVCLLGAGLV DSEVTQTPKYLIKSR KQQAMLRCSPE PGH.......LS VYWYQQAL GQGLQFLIQ YYN....RAE NEKGNIP.D RFSGKQF.SD SSSELNVTSLEL TDSAVYLC ASSQ.. [5.6.4]
TRBV5-9   F           MGCGPVCCVALCLLAAGLV DSGVTQTPRYLIKAR GQRGTLRCSPV SGH.......LS VYWYQQAL GQGPQFLVQ YYR....QEV SGEAQLP.D RFSGKQF.GD FHSELNLTSLEL TDSAVYLC ASSQ.. [5.6.4]
TRBV5-11  P           MGSRLLCCVTLCLLGAGLV DSGVIQTPKYLIKSR KQQGTLRCSLE SGH.......RY VSWYQQAL GQSPQFLVQ YFN....REV NEKGNMP.D *FSGEQF.SD SCSQMNLSSLEL TDSAVYLC ASSQ.. [5.6.4]
TRBV5-12  F           MGSRLLCWVSLFLLGAGLV ESEVTQTPKYLIKSR KEKVTLRCSPK SGH.......RS VYWYQQAL GQGPQFLVQ YYS....QQV SGEAQLP.D RFSGKQF.SD FHSELNLTSLEL TDSAVYLC ASSQ.. [5.6.4]
TRBV5-14  F           MGCSPICCVALCLLAAGLV DSGVIQTPRYLIKAR GQRGTLRCSPV SGH.......LS VYWYQQAL GQGPQFLVQ YYS....QNV YGEAQLP.D RFSGEQF.SD SSSELNLTSLEL TDSAVYLC ASSQ.. [5.6.4]
TRBV5-15  P           MGSRLLCCVTLCLLGAGLV NSGLTLSPKYLIKSR KEQLTLRCSCE SGH.......RS VYWYQQAL GQSPRVLFQ YYD....GKV YQKGNISD* VSGKQFC.DD ARFELSLTSLEL TDSAMYLC ARSQ.. [5.6.4]
TRBV5-16  F           MGSRLLCCVTLCLLGAGLV DSGVTQTPKYLIRSR KQQATLRCSPE SGH.......RY VSWYQQAL GQGPQFLVQ YYD....GKV HTKGNMP.G QFSGEQF.SD SRSQMNLSSLEL TDSAVYLC ASRQ.. [5.6.4]
TRBV5-17  F           MASRFLCCVSLFLLGAGLV ESEVTQTPKYLIKSR KEQVTLRCSPK SGH.......RS VYWYQQAL GQGPQFLVQ YYD....GEI YQKENIS.D RFSGKQF.SD ARSELSLTPLEL TDSAVYLC ASRQ.. [5.6.4]
TRBV5-18  F           MGCGPICCVALCLLAAGLV DSGVTQTPRYLIKAR GQQGTLRCSPV SGH.......LS VYWYQQAL GQGPQFLVE YYN....QDV RGETNLP.D RFSGEQF.SD SSSELNLTSLEL RDSAVYLC ASSQ.. [5.6.4]
TRBV5-19  F           MGSRLLCCVTVCLLGAGLV DSGVTQTPKYLIKSK KQQLTLRCSPE FGH.......RY VSWYQQAL GQGPQFLIQ YYN....REV NEKGNIP.D RFSENQF.SD SRSELNLTSLEL TDSAVYLC ASSK.. [5.6.4]
TRBV5-21  F           MGCGPVCCVALCLLAAGLV DSGVTQTPRYLIKAR GQQGTLRCSPV SGH.......LY VYWYQQAL GQGPQFLVL YYS....QDV YGEAQLP.D RFSGKQF.SD SSSELNLTSLEL TDSAVYLC ASSQ.. [5.6.4]
TRBV5-22  F           MGSRLLCCVTLCLLGAGLV NSGLTVTPKYLIKSR KEHLTLRCSSD SGH.......RS VYWYQQAL GQSPRFLFQ YYD....GKV YQKGNISDK VSGKQFS.DD ARFELSLTSLEL TDSAMYLC ASSQ.. [5.6.4]
TRBV5-24  F           MGCGPVCCVALCLLAAGLV DSGVTQTPRYLIKAR GQRGTLRCSPV SGH.......LS VYWYQQAL GQGPQFLVQ YYS....QDV RGEAQLP.D RFSGKQF.SD SSSELNLTSLEL MDSAVYLC ASSQ.. [5.6.4]
TRBV5-27  F           MGCGPICCVALCLLAAGLV DSGVTQTPRYLIKAR GQRGTLGCSPV SGH.......LS VYWYKLVL GQGPQFLVQ YYS....QDV WGETNLP.D RFSGEQF.SD SSSELNLTSLEL MDSAVYLC ASSQ.. [5.6.4]
TRBV5-28  P           MGSRLLCCVTLCLLGAGLV NSGLTLAPKHLIKSR KE*LTVRCSPE SGY.......RS VY*YQQAL AQGPQFLVQ YYD....GEI YQKGNIS.N RVSGKLF.SD ACSELSLTPLEL THSAVYLC ASSQ.. [5.6.4]
TRBV5-29  F           MGSRLLCCVTLCFLGGGLV DSGVTQIPKYLIKSR KQQVTLRCSPE SGH.......RY VSWYQQAL GKSPQFLVQ YYD....GKV RTKGNMP.D RFSGEQF.SD SRSQLNLSALEL TDSAMYLC ASRQ.. [5.6.4]
TRBV5-30  F           MGSRLLPWVMLYLLGAGPL EAEVTQTPRHLIKTK GQTATLRCSPM SGH.......SS VSWYQQAR SQGPQFLFE FYE....TLQ RDKGNFS.N RFSAKQF.RD FSSELNVNFLEL TDSALYLC ASSL.. [5.6.4]
TRBV6-1   F           MSPCLLGCVVFCLLQAGAV HAGVTQDPRFQVVRT GQRVTLRCTQD LNY.......DY MYWYRQDP GHGLRLIHY SDG....PPA TEKGDVP.D GYNVSRP.ST EDFPLTLESASR SQTSVYFC ASSY.. [5.6.4]
TRBV6-2   P           TSPCLLGCVVFCLLQAGAV HAGVTQDPRFQVVKI GQSATLNCTQD LGH.......NY MYWY*QDP GHGLRLIHY SAG....PPT TQQGDVP.E GYSVSRS.NK ENFPLTLESANP SQTSVYFC ASSY.. [5.6.4]
TRBV6-3   P           MSPCLLDCVVFRLLQAGVV HAGVTQDPRFQVVRT GQRATLTCTQD LGH.......DR MY*YQQDL AHGLRLIHY SAG....MHT SKPGGVP.D GYNASRS.NT ENFSLTVESANH SQTSAYFC ASSY.. [5.6.4]
TRBV6-4   P           MSPCLLGCVVFCLLQAGAV HAGVTQDSRFQVVTT GQRETLKCTQD LNH.......DS MYWC*QDL GHGLRLMRY SAG....PPT TEQGDVP.E GYSISIS.SR ENFPLTLEFANP SQTSVYFC ASSY.. [5.6.4]
TRBV6-5   F           MSPCLLGCVVFCLLQAGAV HAGVTQDPGFQVVRT GESATLRCTQD LSF.......SY MYWYRQDP GHGLRLIHC SVI....PPA TEKGAVP.E GYSVSRP.ST ENFPLTLESANA SQTSVYFC SCGY.. [5.6.4]
TRBV6-6   F           MSPCLLGCVVFCLLQAGVV HAGVTQDPRFQVMRT GQNVTLKCTQD LGH.......DP MYWYRQDP GPGLRLIHY SAG....PPT MVQGDVP.D GYSVSRS.SK ENFPLMLESANL SQTSVYFC ASSY.. [5.6.4]
TRBV6-11  F           MSPCLLGCVVFCLLQAGVV HAGVTQDPRFQVVRT GQRVTLRCTQD LSH.......DY MYWYRLDL GHGLRLIHY SAA....APN TEKGDVP.K GYSISRP.ST EDFPLTLESANR SQTSVYFC ASSY.. [5.6.4]
TRBV6-13  P           MSPCLLGCVVFCLLQAGVV HAGVTQAPRFQVVTT GQRVTVTCTQD LGH.......DP MYWY*QDL GHGLRLIHY SAG....PPS TEKGDVP.D GYRVSRS.SK ENFPLTLESASR SQTSVYFC ASSY.. [5.6.4]
TRBV6-16  F           MSPCLLGCVVFCLLQAGAV RAGVTQDPRFQVVRT GQNVTLKCTQD LGH.......NS MYWYRQDP GHGLRLIHY SRG....PPS TERGDVP.D GYSVSRS.SK ENFPLTLESANP SQTSVYFC ASSY.. [5.6.4]
TRBV6-17  ORF         MSPCLLGCVLFCLLQAGVV HAGVTQAPRFQVVRT GQNATLTCTQD LGH.......DP MYWYRQDL GHGLRLIHY SAG....PPS TEKGDVP.D GYRVSRS.SK ENFPLTLESASR SQTSVYFC ASSY.. [5.6.4]
TRBV6-18  F           MSPCLLGCVVFCLLQAGAV HSGVTQVPRFQVVRT GQRVTLKCTQN LDH.......NS MYWYRQDP GNGLRLIHY SRG....PPT TEKGDMP.D GYSVFRS.SK ENFPLMLESANP SQTSVYFC ASSY.. [5.6.4]
TRBV6-19  F           MSPCLLGCVVFCLLQAGAV HAGVTQDPGFQVVRT GESATLRCTQD LSF.......SY MYWYRQDP GHGLRLIHY SVI....PPA TEKGAVP.E GYSVSRP.ST ENFPLTLESANA SQTSVYFC SCGY.. [5.6.4]
TRBV6-20  ORF         MSPCLLGCVVFCLLQAGAV HAGVTQDPRFQVVRT GQSTTLKCTQD LGQ.......NS MYCYRQDL GLGLRLIHY SAA....YPT TVKGDVP.E GYSVSRP.ST EDFPLTLKSANR SQTSVYFC ASSY.. [5.6.4]
TRBV6-21  ORF         MSPCLLGYVVFCLLQAGAV HAGVTQDPGFQVVRT GQKATVTCTQD LGH.......DR MYWNQHDL GHR..LIQY SVG....AST SEPGDIP.E GYSFSRS.NT DYFSLTLESASS SRHVCTSV PAVT.. [5.6.4]
TRBV6-22  P           MSPCLLGCVVFCLLQAGVV HAGVTQAPRFQVVTT GQRVTVTCTQD LGH.......DP MYWY*QDL GHGLRLIHY SAG....PPS TEKRDVP.D GYRVSRP.SK GNFPLTLESASR SQTSVYFC ASSY.. [5.6.4]
TRBV6-23  ORF         MSLCLLGCVVFCLLQAAAV HAGVTQDPRFQVMTT GQSMTLKCTQD LDH.......NS MYWYRQDP GHGLRLIHY SRG....PPS TERGDVP.D GYSVSRS.SK ENFPLTLESANP SQTSVYFC ASSY.. [5.6.4]
TRBV6-24  P           MSLCLLGCVVFSLLQAGLI HAGVTQDPRFQVVKT GQSTTLKCTQD LGH.......NS MYWYRQDP GLGLRLIHY *AA....YPS TEKGDVP.E GYNVSRP.ST EDFPLTLKSASR SQTSVYFC ASSY.. [5.6.4]
TRBV6-25  P           MSPCLLGCVVFHLLQAGPI HAGVTQDPRFQVVRT GQRATLECTQD LGH.......DR MY*YGKDM GPGLRLICY SAG....PPT TEKGDVP.D GYRVSRS.SK ENFPLMLESASR SQTSVCFC ASSY.. [5.6.4]
TRBV6-26  F           MSPCLLGCVVFCLLQAGAV HAGVTQAPRFQVMRT GQNATLKCTQD LGH.......DC MCWDQQDL GHGLRLIHH SAG....PPT TEKGDVP.D GYRVSRS.SK ENFPLTLESASH SQTSVYFC ANSY.. [5.6.4]
TRBV6-27  F           MSPCLLGCVVFCLLQAAVV HAGVTQDPRFQVVRT GQNATLYCTQD LGH.......DR MFWYRQDL GHGLRLIHY SAA....YPS TEKGDVP.E GYNVSRP.ST EDFPLTLKSASR SQTSVYFC ASS*.. [5.6.3]
TRBV6-28  F           MSPCLLGCVVFCLLQAGAV HAGVSQDPRFQVVRT GQNATLKCTQD LGH.......DG MYWY.QDL GHGLRLIHY SAG....PPS TEKGDVP.D GYRVSRS.SK ENFPLTLESASR SQTSVYFC ASSY.. [5.6.4]
TRBV6-29  F           MSLCLLGCVVFCLLQAGVV HAGVTQDPRFQVVRT GQRATLKCTQD LNH.......DS MYWYRQDP GHGLRLIHY SSF....TDA TYKGDVP.D GYNVSRS.ST ENFPLTLESANP SQTSVYFC ASST.. [5.6.4]
TRBV7-1   F          MGSRLLCWATLCLLGVGHTG AGVVSQSPRHRVAGR GQNVTLRCDPI SGH.......IS LYWYRQTL GQGPEFLTY FQN....EQE LDKSGMPKN RFSAKRP.QS TYSYLNIQPAEP GDSAVYLC ASSP.. [5.6.4]
TRBV7-2   F          MGSRLLCWVTLCVLGVGHTR AGVVSQSPGYRVTGR GQNVTLRCDPI PGH.......QV LYWYRQML GQGPEYLTY FQG....KEE PDKSGMPKN RFSAERP.ES TYSYLKIQPVEP KDSAVYLC ASSP.. [5.6.4]
TRBV12-1  F         MGTQALCCIALCLLGVGTTDA NLGVTQTPRHEVTEK GQAVTLSCEPI KSH.......TA LYWYRQTS VRGLELLIY FRS....EAP VDESGMPKD RFSAKMP.NA SFSTLKIQPTDP GDSATYLC ASSK.. [5.6.4]
TRBV12-2  F           MATKLLFCVALCLLGVGPT DAGVTQTPRHEVTEK GQAVTLSCEPI KSH.......AA LFWYRQTS VRGLELLIY FNN....QSP VDESGMPKD RFSAKMP.NA SFSTLKIQPTEP GDSATYLC ASSK.. [5.6.4]
TRBV14    P           VVSRLLRLASLCLLGARCI SAEVAQSPSHRVIEK KQAVNLSCDPV SGH.......NS LFWYYHAV GTEMKLLIY FLR....ESV QDNSGMPKG RFTAERT.QG TSSTLRVHSAEL GDSGVYFC ASSR.. [5.6.4]
TRBV15    F           MEPRLLLCVALCLLGAGHV GAMVIQNPRYLVTGL GKPVTLSCSQD MKH.......DV MYWYQQKP SQAPKLLLY YYD....KEI TREKNTS.E NFQSSRP.NT SFCSLDIRSAGL GDSAVYLC ASSR.. [5.6.4]
TRBV16    F           MSPTLTCFTVLCLLGAGFL EAEVTQTPGHLVQGK GLEVEMYCVPK KGH.......IY VFWYQQIL AKEFKFLIS FQN....DKV LDDKEMPKK RFSAKCP.SN SPCSLKIQPAEP QDSAMYFC ASSD.. [5.6.4]
TRBV19    F           MCNQVICCVALCLFGAGTV AGEVTQTPKYLLKKE GGAVTLECEQD FDY.......DS MYWYRQDP GLGLRRIFF SQV....VKD VQEEDIA.K GYSASREK.K PFFPLTVTSAQK NQTALYLC AASR.. [5.6.4]
TRBV20    F                MLALLLLLGPSSGL GAVVSQQPRRAVSKS GASVTIECRAV DFQ......ATT VFWYRQFP KRGLVLMAT SNAG...TDA TYEQGYNKD KLPISQP.NL TFSSLMVMNVDP ADSSLYFC GAR... [6.7.3]
TRBV21-1  F         MCFSLLCYVAFFFFFWGAGSM DTEVTQSPSHLVKGK DQKAKMDCVPI KGH.......SY VYWYRKKP EGAFEFLVY LQN....QDV VEDTAMFKQ QFSADCP.PN SPCSLEIKSTKA ADSALYFC ASSQ.. [5.6.4]
TRBV21-2  F          MCLSLLGCVALFLWGAGSM DTEVTQNPSHLVKGK DQRAKMDCVPI KGH.......SN VYWYRKKL EAFEFLVYL WNG.....KI TEDTAMFKQ RFSAECP.QN SPCSLEIKSTEA PDSALYFC ASS*.. [5.5.3]
TRBV21-3  F          MCLGLLCCVALLFWGAGSM DIEVTQSPSHLVKGK DQRAKMYCVPK KGH.......SY VYWYRRKL EEAFEFLVY LQD....EKI IEDTDMFKQ RFSAECP.QT SPCSLGIKSTKA SDSALYFC ASSQ.. [5.6.4]
TRBV21-4  F          MCFSLLCWAAFFFFWGAGSM DTEVIQSPGHLVKGK DQKAKMDCVPI KGH.......VH VYWYRKKP EGAFEFLVY LQN....QDI VEDTEVFQQ QFLAQWP.QN SPCSLEIKSTKA ADSALYFC ASSQ.. [5.6.4]
TRBV21-5  F          MCLGLLCCVALFIWGAGSM DAEVTQSPSHLVKGK GQKAKMDCVPI KGH.......IN VYWYRRKL EEALEFLVY LQK....QDI VEDTEVFKE QFSAQCP.QN SPCSLEIKSTKA ADSALYFC ASSQ.. [5.6.4]
TRBV21-6  F          MCLGLLCCVVLFFWGAGSM DTEVTQRPSHLIKGK DQKAKMDCVPK EGH.......PY VYWYRKKL EGAFEFLVY FQN....QDT MEDTVEFKQ RFSAECP.QN SPCSLEIKSTEA ADSALYFC ASSQ.. [5.6.4]
TRBV22    F             MGSWALYLALCLLGAGP KDAEIYQIGFLLAEA GQDVTLECKRN LGY.......SA MYWYQQDP GQGLKLTYY STL....ETD IQRGDLS.E GYSVSRE.KK ELFPLTVKSAHT KQTAVYLC SGA... [5.6.3]
TRBV24    F           MGSVLLCCVVFCLLGTGSM DTGVTQTPRNRIAVT GKSTVLECSQT KGH.......ES MYWYRQDP GRGLRLVYS SYD....ING INKGDVS.A GYNVSRK.EK AKFSLYLEPATP NQKAIYFC ASSI.. [5.6.4]
TRBV25    F           MAVRLLCCVTLYLLGAGFT DADVYQTPRHCVTGT GRKITLECSQT MGH.......DN MYWYQQDP GMELQLIHY SYG....VNT TEKGERS.S RSTVSRI.SK EHFPLTLETASS SQTSSYFC ASSD.. [5.6.4]
TRBV26    F           MRNRLLCWVVLLLLRIGLE DAAVIQFPRHRILKA EKEFTLQCSQK MNH.......YA MYWYRQDP GFGLQLIYY STG....PDT FKKGDVP.E GYHVSRD.EL DHFPLTLKSASP NQTSVYFC ASTE.. [5.6.4]
TRBV27    P           LGPRLLGCVLFCFLGAGPL EADVTQSPRHRITET RKRVTLTCSQN MNH.......DV MYWYRQDP GLGPKLVYF SRN....VGF VEKGDIS.D GYTASRE.EK PNFPLILELTST NQTSLYLC ASSE.. [5.6.4]
TRBV28    F            MGIRLLYAFCFLGVGLM DAQVTQTPRYLVKRR GEKVVIECMQN MDH.......ER MFWYRQDP ALGLRLLHF SIN....VDV LEEGDVP.Y GYSVSRT.KK KSFPLTLESATT NQTAMYLC ASSE.. [5.6.4]
TRBV29    F               MLTLLLLLLGLGSVF GALLSQKPSRAIRQR GTSMTIECQAD SQL.......NF MYWYRQLP GQSLVLMAT ANQ....GSK ATYESEFTE DKFPISRPNL AFSTLTVSNASS EDSSSYFC SAG... [5.6.3]
TRBV30    F             MPCCLLALLLGTFLGVR AQTIHQWPSTRVQPA GSPLSLECTVK GTS......SPN LYWYRQEA GGSLQLLFS SVG.....VD QIEPREF.Q NFEASRPQ.D GQFILSSKKLQL NNSGFYLC AWS... [6.5.3]
```
