# Supplementary figures and images for: The expansion of the TRB and TRG genes in domestic goats (Capra hircus) is characteristic of the ruminant species
Source: BMC Genomics. 2020 Sep 11;21:623. doi: 10.1186/s12864-020-07022-x (PMC7488459; doi:10.1186/s12864-020-07022-x)

A

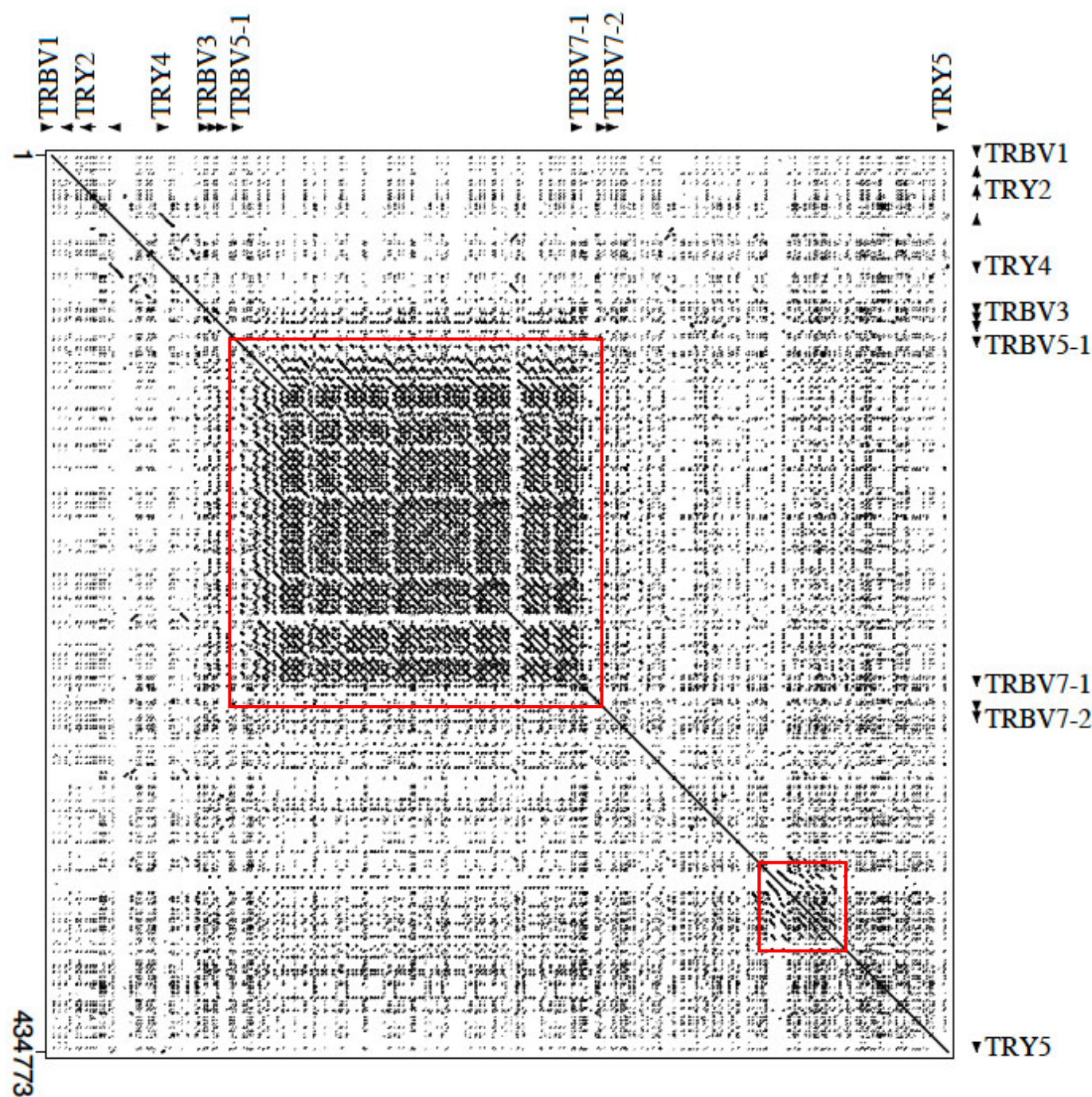

Supplement: Supplementary file 5 — Additional file 5: Figure S2. (A) Dot-plot of the goat TRBV cluster sequence against itself. Description: With the exception of the main diagonal line for the match of each base with itself, dots and diagonal lines indicate internal homology units in the sequence. The red boxes show the TRBV regions underwent to duplication events. [file 12864_2020_7022_MOESM5_ESM.pdf]

B

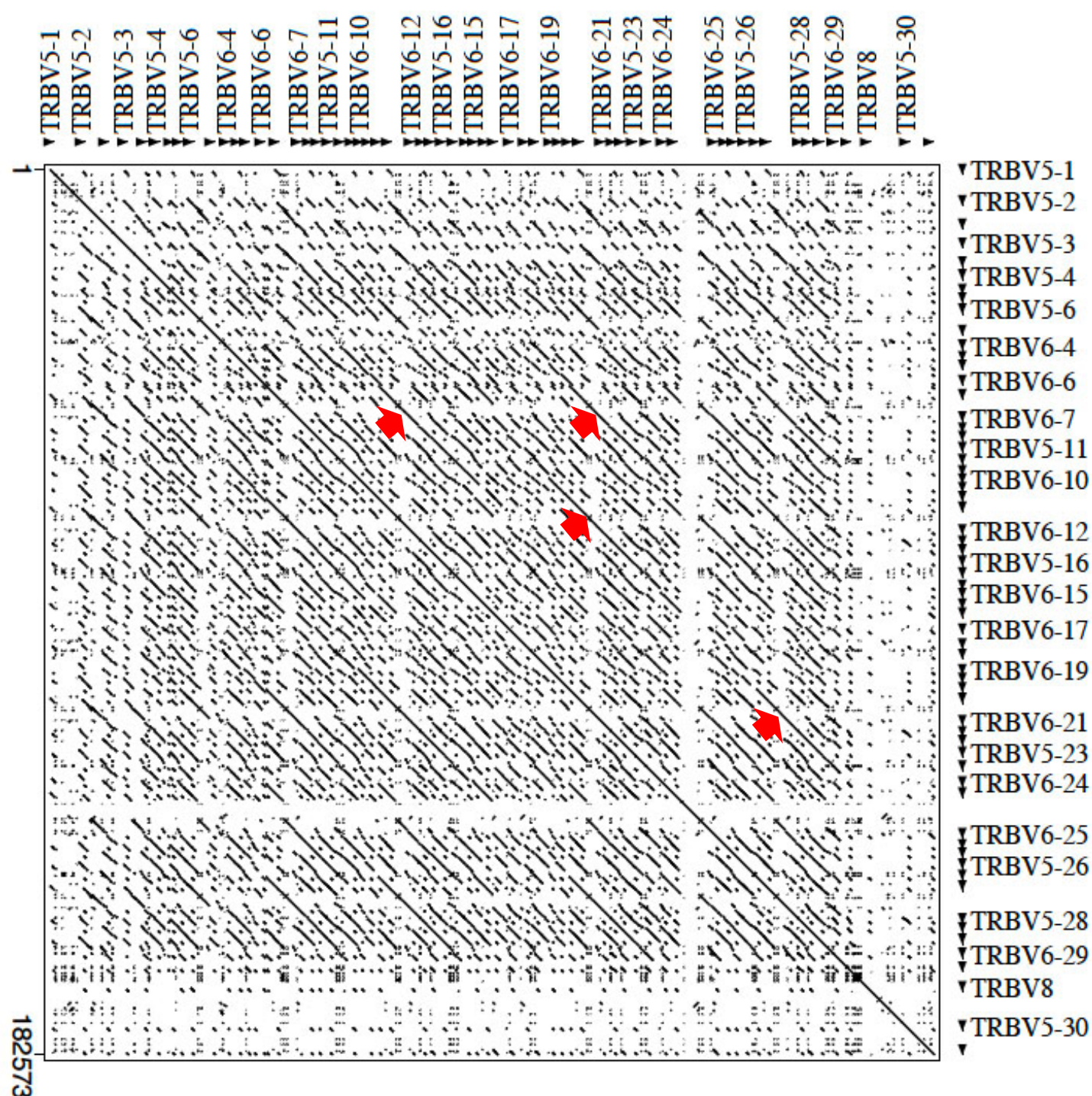

Supplement: Supplementary file 6 — Additional file 6: Figure S2. (B) Dot-plot of the goat TRBV cluster sequence against itself. Description: Enlargement of the wider red box in (A) showing the pattern of parallel lines due to the tandem duplicative events of the TRBV5 and TRBV6 genes. The arrows indicate long homology lines determined by a repetitive TRBV5-TRBV6 base unit. [file 12864_2020_7022_MOESM6_ESM.pdf]

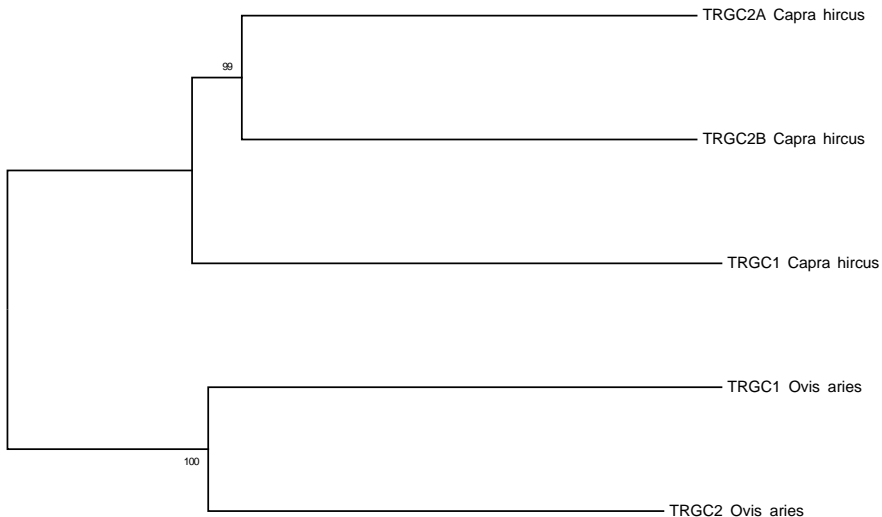

0.0050

Supplement: Supplementary file 12 — Additional file 12: Figure S4. The NJ tree inferred from the genomic sequences of the goat TRGC1, TRGC2A and TRGC2B cassettes, together with the sheep TRGC1 and TRGC2 cassette sequences. Description: The evolutionary analysis was conducted in MEGA7 [35]. The optimal tree with the sum of branch length = 0.08464556 is shown. The percentage of replicate trees in which the associated taxa clustered together in the bootstrap test (100 replicates) is shown next to the branches [36]. The tree is drawn to scale with branch lengths in the same units as those of the evolutionary distances used to infer phylogenetic trees. The evolutionary distances were computed using the p-distance method [37] and are in the units of the number of base differences per site. The analysis involved 5 nucleotide sequences. All positions containing gaps and missing data were eliminated. There were a total of 17,718 positions in the final dataset. [file 12864_2020_7022_MOESM12_ESM.pdf]

J-RS

J-NONAMER

J-HEPTAMER

Goat

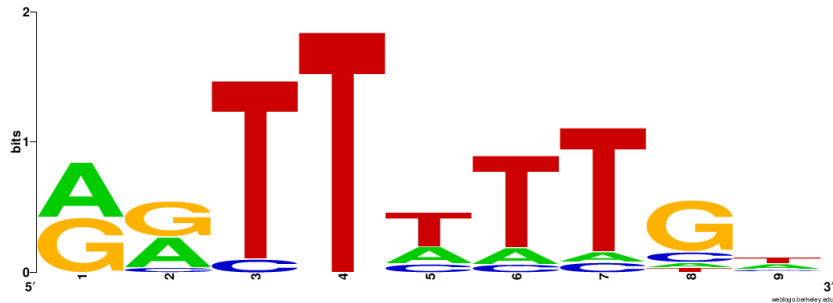

12

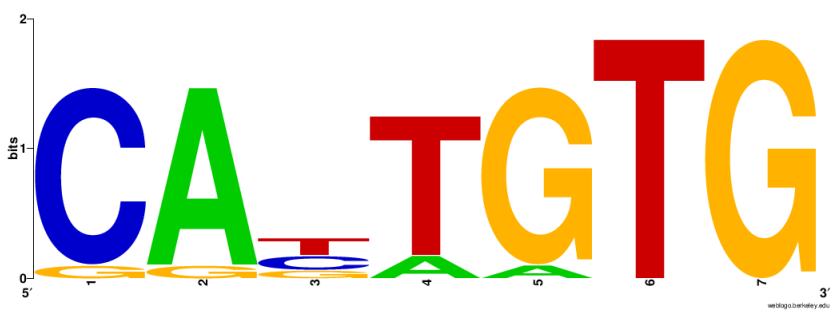

Sheep

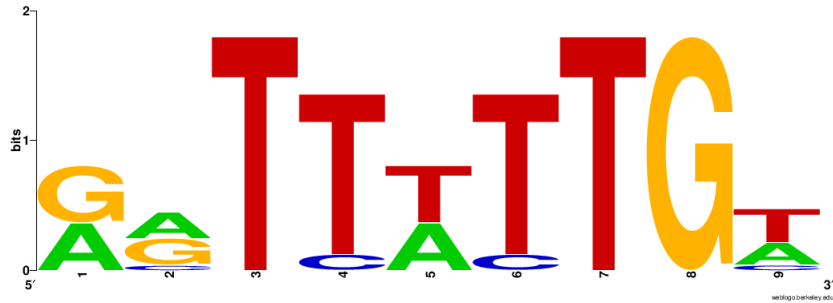

12

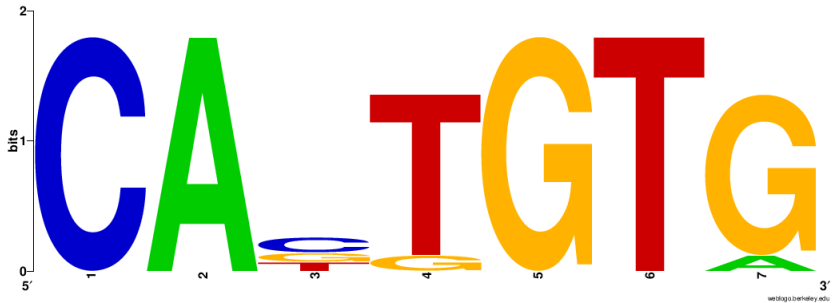

Supplement: Supplementary file 17 — Additional file 17: Figure S7. J-RS sequence logos. Description: Recombination signal sequence logos of the heptamers and nonamers of the goat and sheep TRGJ genes generated using Weblogo [55] The height of letters indicates the relative frequency of each nucleotide at that position. [file 12864_2020_7022_MOESM17_ESM.pdf]

A

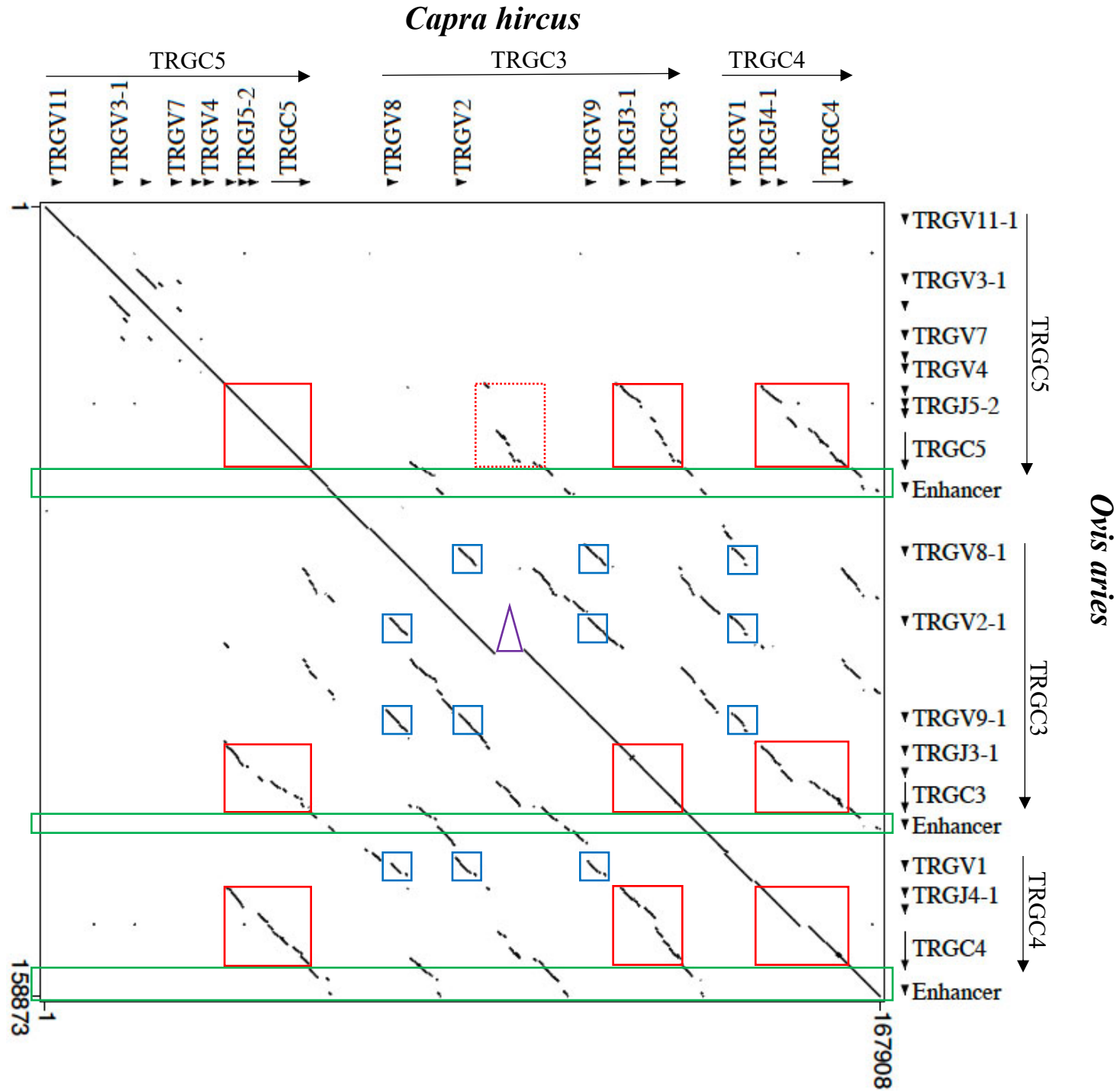

Supplement: Supplementary file 18 — Additional file 18: Figure S8. (A) Dotplot matrix of goat/sheep TRG1 genomic comparison. Description: The transcriptional orientation of each gene is indicated by arrows and arrow-heads. The triangle indicates a gap (insertions or deletions) between the homology unit. Coloured boxes enclose J-C blocks (red), TRGV genes (blue) and enhancer-like sequences (green). [file 12864_2020_7022_MOESM18_ESM.pdf]

*Capra hircus*

**B**

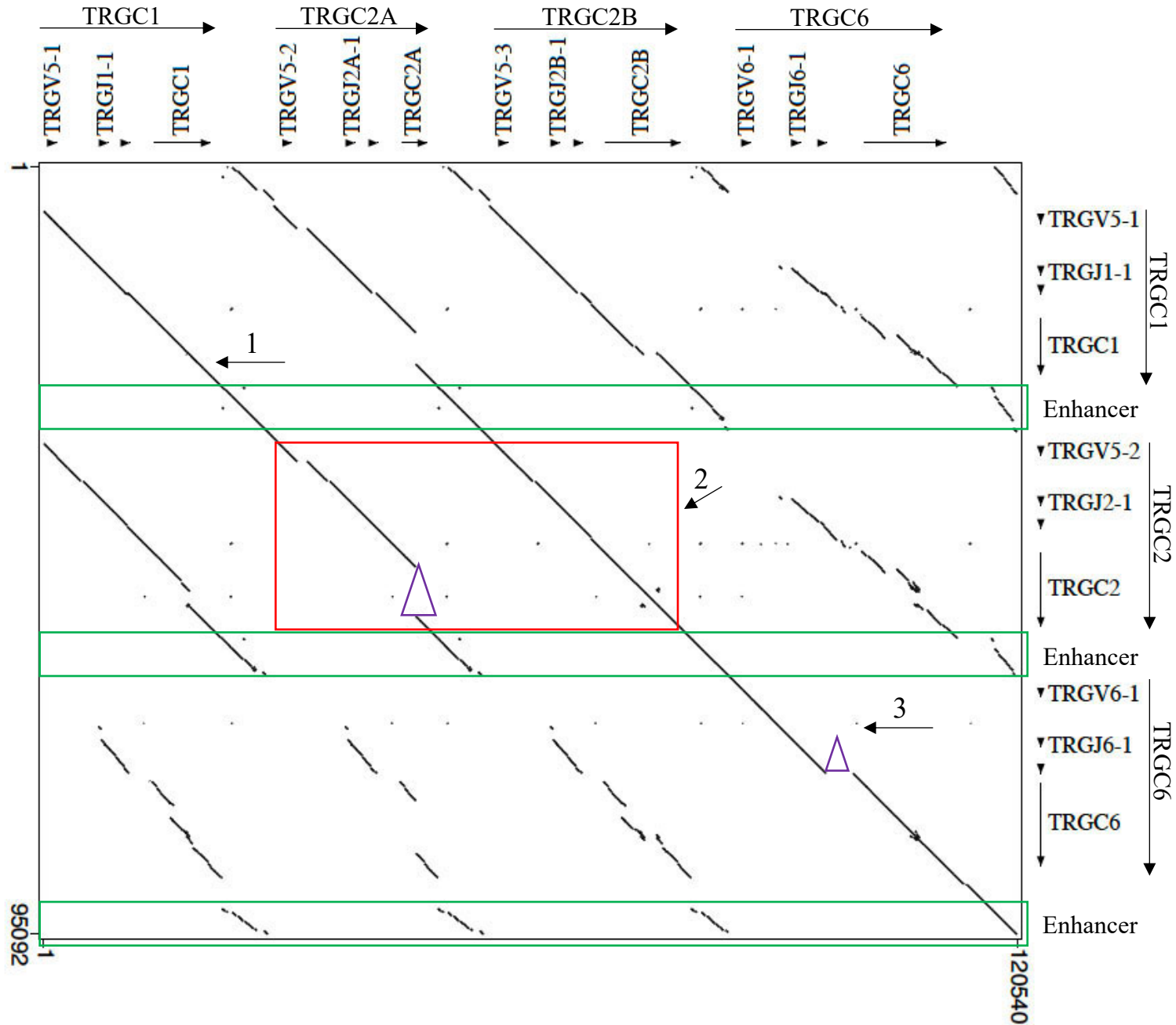

*Ovis aries*

Supplement: Supplementary file 19 — Additional file 19: Figure S8. (B) Dotplot matrix of goat/sheep TRG2 genomic comparison. Description: The transcriptional orientation of each gene is indicated by arrows and arrow-heads. The triangle indicates a gap (insertions or deletions) between the homology unit. The regions of more extensive homology are numbered. The parallel lines representing the duplication of TRGC2A and TRGC2B cassettes are boxed (red). Green rectangle enclose the position of the enhancer-like sequences in the sheep locus. [file 12864_2020_7022_MOESM19_ESM.pdf]
