# Supplementary material for: The expansion of the TRB and TRG genes in domestic goats (Capra hircus) is characteristic of the ruminant species
Source: BMC Genomics. 2020 Sep 11;21:623. doi: 10.1186/s12864-020-07022-x (PMC7488459; doi:10.1186/s12864-020-07022-x)
Supplement: Supplementary file 7 — Additional file 7: Figure S3. (A) Description of the goat TRBD genes. The inferred amino acid sequence of the TRBD genes in the three coding frames are reported. [file 12864_2020_7022_MOESM7_ESM.pdf]

(A)

TRBD gene name	5’D-NONAMER	5’D–SPACER	5’D-HEPTAMER	D-REGION	3’D-HEPTAMER	3’D-SPACER	3’D-NONAMER
	<u>GGTTTTTGT</u>	************	<u>CACTGTG</u>		<u>CACAGTG</u>	***********************	<u>ACAAAAACC</u>
TRBD1	cgtttttgt	ataaagctataa	cgctgtg	GGGACAGGGGGGC<br>G T G G<br>G Q G G<br>D R G	cacggtg	attcaaccctatgggaatccttt	acaaaaacc
TRBD3	catttttgt	atcacagtgtaa	cattgtg	GGGGCTGGGGGGGTGGG<br>G A G G V<br>G L G G W<br>G W G G G	cacaatg	attcagttagaggaagtgctttt	acaaaaagc
TRBD2	catttttgt	atcacagtgtaa	cattgtg	GGACTTTGGGGGGGC<br>G L W G G<br>D F G G G<br>T L G G	cacgatg	attcagttagaggaagtgctttt	acaaaaagc
