# Supplementary material for: The expansion of the TRB and TRG genes in domestic goats (Capra hircus) is characteristic of the ruminant species
Source: BMC Genomics. 2020 Sep 11;21:623. doi: 10.1186/s12864-020-07022-x (PMC7488459; doi:10.1186/s12864-020-07022-x)
Supplement: Supplementary file 8 — Additional file 8: Figure S3. (B) Description of the goat TRBJ genes. The donor splice site for each TRBJ is shown. The canonical FGXG amino acid motifs are underlined. The TRBJ1–3 and TRBJ2–7 ORF and TRB3–6 pseudogene are indicated in italics. [file 12864_2020_7022_MOESM8_ESM.pdf]

(B)

TRBJ gene name	J-NONAMER GGTTTTTGT	J-SPACER ************	J-HEPTAMER CACTGTG	J-REGION	5'splice donor
TRBJ1-1	tttcctcct	tgctccgcttca	ctgtgtg	AACACTGAGGTTTTCTTTGGAAAAGGCACCAGGCTCACGGTTGTAG<br>N T E V F F G K G T R L T V V	gtaaga
TRBJ1-2	cgttgtagg	gtggctgtgttc	tgatgtg	TTATGAATATCACTTCGGCCCAGGGACCAAGCTGACAGTTGTAG<br>Y E Y H F G P G T K L T V V	gtaagg
TRBJ1-3	*gttttgaag*	*tgaatcctggag*	*gcttggc*	*TTTGGAGACTTCTACTTTTTGAGGGGGGAGGGAAGACGGCTCGCTGTT*<br>*F G D F Y F L R G E G R R L A V*	*gtagat*
TRBJ1-4	tttccctac	caggctttaggg	ttgtgtg	ACTAATGAAAGACTCTATTTCGGCAATGGGACGAAGCTTTCCGTCTTGG<br>T N E R L Y F G N G T K L S V L	gtgagt
TRBJ1-5	gggtttgcc	acacccgcgttg	ggccgtg	GAGCAACCAGGCACAGCACTTTGGACACGGGACCTGGCTCGCCGTCCTGG<br>S N Q A Q H F G H G T W L A V L	gtaaaa
TRBJ1-6	ggtttcgcc	acagccacctgc	agctgtg	GGCCTATAATTCGCCCCTCCACTTTGGGATCGGCACCAGGCTCACCGTGACAG<br>A Y N S P L H F G I G T R L T V T	gtatcg
TRBJ3-1	gaattcttg	gcagccccttcc	cactgtg	CTCCTATGATGAGCTGCACTTCGGGCCAGGGACCAGGCTCACCGTGCTAG<br>S Y D E L H F G P G T R L T V L	gtaaga
TRBJ3-2	agtttgcgc	ctgggttcccag	ggctgtg	TGAACGCCGCGCAGCTGTACTTTGGAGCTGGTTCCAAGCTGACTGTGCTGG<br>N A A Q L Y F G A G S K L T V L	gtaagg
TRBJ3-3	ggtttttgt	cctgggtccccg	ggctgtg	AGCCAAAGCACTCAGTACTTCGGCGCGGGCACTCGGCTGTCGGTGCTAG<br>S Q S T Q Y F G A G T R L S V L	gtgagc
TRBJ3-4	ggtttttgc	gcggggctgggg	ggccgtg	ACTCAGAGACGCAGTACTTCGGGCCGGGCACGCGGCTCCTGGTGCTAG<br>S E T Q Y F G P G T R L L V L	gtgagt
TRBJ3-5	ggtttgctt	gcggggctggga	ctctgtg	AGCCAGTGAGCGGTATTTCGGCGCCGGCACCAGGCTCACGGTCACAG<br>A S E R Y F G A G T R L T V T	gtgaga
TRBJ3-6	*cgggcggcg*	*cgcctcgattct*	*ttgtggg*	*GCGCCGCGGCGCCGCCCTGACCTATGGGGCCGGCAGCGGGCTGGCCGCGGTGG*<br>*R R G A A L T Y G A G S G L A A V*	*gcgaga*
TRBJ2-1	gaattcttg	gcagccccttcc	cactgtg	CTCCTATGGGGAGCTGCACTTCGGGTCAGGGACCAGGCTCACCGTGCTAG<br>S Y G E L H F G S G T R L T V L	gtaaga
TRBJ2-2	agtttgcgc	ctgggtccccag	ggctgtg	TGAACACAGATCCCCTTTACTTTGGAGCTGGTTCCAAGCTGACTGTGCTGG<br>N T D P L Y F G A G S K L T V L	gtaagg
TRBJ2-3	agtttttgt	cctgagcctccg	ggctgtg	AGCAATAACCCTCTGCATTTCGGAGGGGGCACTCGGCTGTTGGTACTAG<br>S N N P L H F G G G T R L L V L	gtaagt
TRBJ2-4	tatttctgt	gctgagccctga	ggctgtg	AGTACAGACACGCAGTACTTCGGCCCGGGAACCCGGCTGTCGGTGCTAG<br>S T D T Q Y F G P G T R L S V L	gtgagc
TRBJ2-5	ggtttttgc	gcggggctgggg	ggccgtg	ACTCAGACCCAGTACTTTGGGCCTGGCACGCGGCTCCTGGTGCTAG<br>T Q T Q Y F G P G T R L L V L	gtgagt
TRBJ2-6	gatttttgt	ggggctccccgg	ggctgtg	CTTTGCAGGCGCCGCCCTGACCTTCGGGGCCGGCAGCCGGCTGACGGTGGTGG<br>F A G A A L T F G A G S R L T V V	gtgagt
TRBJ2-7	*gtttgagtg*	*ccgggctcttcc*	*tccgtgc*	*TCCTATGAGCAGTATTTCGGCCCAGGCACCAAGCTCACGGTCGTAG*<br>*S Y E Q Y F G P G T K L T V V*	*gtgaga*
