# Supplementary material for: The expansion of the TRB and TRG genes in domestic goats (Capra hircus) is characteristic of the ruminant species
Source: BMC Genomics. 2020 Sep 11;21:623. doi: 10.1186/s12864-020-07022-x (PMC7488459; doi:10.1186/s12864-020-07022-x)
Supplement: Supplementary file 9 — Additional file 9: Figure S3. (C) Description of the goat TRBC genes. The IMGT Protein display of the goat TRBC gene compared with the human, sheep, pig and dromedary Cβ proteins. The descriptions of the strands and loops were collected according to the IMGT unique numbering for the C-DOMAIN [54]. [file 12864_2020_7022_MOESM9_ESM.pdf]

(C)

```
                                                                                           C-DOMAIN
                                                                                            [EX1]

                               A        AB      B           BC       C     CD      D          DE           E      EF   F                FG                  G
                             (1-15)          (16-26)     (27-38)  (39-45)       (77-84)                 (85-96)     (97-104)         (105-117)          (118-128)
                          --------------->  ---------->            ------>       ------->              ----------->  ------->                           ----------->
                          1        10  15   16    23 26 27      36 3941 45       77 80 84              85  89    96  97   104 105                   117 118121  128
                  87654321|........|....|123|......|..| |........| |.|...|1234567|..|...|12345677654321|...|......|12|......| |......123456787654321.....| |..|......|
Caphir TRBC1     (D)NLSRVHPPKVAVFEPSEAEISR..TQKATLVCLAT GFYP..DHVE LTWWVNRKQVTT..GVSTDPEPYKEDLTQ.NDSRYCLSSRLRVTAAFWH.NPRNHFRC QVQFYGLTDQ..DQWEEQDRAKPVTQNI SAETWGRA...
Caphir TRBC3     (D)DLSRVHPPKVAVFEPSEAEISQ..TQKATLVCLAT GFYP..DHVE LTWWVNRKQVTT..GVSTDPEPYKEDLTQ.NDSRYCLSSRLRVTAAFWH.NPRNHFRC QVQFYGLTDQ..DQWDEQDRDKPVTQNI SAETWGRA...
Caphir TRBC2     (D)DLRQVHPPKVAVFEPSEAEISR..TQKATLVCLAT GFYP..DHVE LTWWVNRKQVTT..GVSTDPEPYKEDLTQ.NDSRYCLSSRLRVTAAFWH.NPRNHFRC QVQFYGLTDQ..DQWEEQDRDKPVTQNI SAETWGRA...
Oviari TRBC1     (D)NLSRVHPPKVAVFEPSEAEISR..TQKATLVCLAT GFYP..DHVE LTWWVNRKQVTT..GVSTDPEPYKEDLTQ.NDSRYCLSSRLRVTAAFWH.NPRNHFRC QVQFYGLTDQ..DQWEEQDRDKPVTQNI SAETWGRA...
Oviari TRBC3     (D)DLRQVHPPKVAVFEPSEAEISR..TQKATLVCLAT GFYP..DHVE LTWWVNRKQVTT..GVSTDPEPYKEDLTQ.NDSRYCLSSRLRVTAAFWH.NPRNHFRC QVQFYGLTDQ..DQWEEQDRDKPVTQNI SAETWGRA...
Oviari TRBC2     (D)DLRQVHPPKVAVFEPSEAEISR..TQKATLVCLAT GFYP..DHVE LTWWVNRKQVTT..GVSTDPEPYKEDLTQ.NDSRYCLSSRLRVTAAFWH.NPRNHFRC QVQFYGLTDQ..DQWEEQDRDKPVTQNI SAETWGRA...
Susscr TRBC1     (E)DLQQVRPPKVAVFEPSEAEISR..TQKATLVCLAT GFYP..DHVE LSWWVNGKQVQS..GVSTDLQPYREDPSR.NDSSYCLSSRLRVTAAFWH.NPRNHFRC QVQFYGLTED..DEWEY.NWTKPITQNI SAEAWGKA...
Susscr TRBC3     (E)DLQQVRPPKVAVFEPSEAEISR..TQKATLVCLAT GFYP..DHVE LSWWVNGKQVQS..GVSTDLQPYREDPSR.NDSSYCLSSRLRVTAAFWH.NPRNHFRC QVQFYGLTED..DEWEY.NWTKPITQNI SAEAWGKA...
Susscr TRBC2     (E)DLQQVRPPKVAVFEPSEAEISR..TQKATLVCLAT GFYP..DHVE LSWWVNGKQVQS..GVSTDLQPYREDPSR.NDSSYCLSSRLRVTAAFWH.NPRNHFRC QVQFYGLTED..DEWEY.NWTKPITQNI SAEAWGKA...
Camdro TRBC1     (E)DLSQVKPPKVVVFEPSEAEISR..TQKATLVCLAT GFYP..DHVE LSWWVNGKEVQS..GVSADPQPYREDPNL.SDSTYCLSSRLRVSATFWH.NPRNHFRC QVHFHGLTDKDRDQWKE.GWPMPISQNV SAETWGRA...
Camdro TRBC3     (E)DLSQVKPPKVAVFEPSEAEISR..TQKATLVCLAT GFYP..DHVE LSWWVNGKEVQS..GVSTDPQPYREDPSL.SDSTYCLSSRLRVSATFWH.NPRNHFRC QVHFHGLTDKDRDQWKE.DWPMPVSQNV SAEAWGRA...
Camdro TRBC2     (E)DLSQVKPPKVAVFEPSEAEISR..TQKATLVCLAT GFYP..DHVE LSWWVNGKEVQS..GVSTDPQPYREDPSL.SDSTYCLSSRLRVSATFWH.NPRNHFRC QVHFHGLTDKDRDQWKE.DWPMPVSQNV SAEAWGRA...
                  : :* .* **:*.**********.  *********** **:*  **** *:**** *:* :  ***:* :* .*:    .** *********:*:**: ******** **:*:**::.  *:*   ..  *::* : ***:**.*
```

```
                        CONNECTING-REGION     | TRANSMEMBRANE-REGION |CYTOPLASMIC-REGION

                     [EX2]                 [EX3]                     [EX4]
Caphir TRBC1      (D)CGVTS  (A)SYQQGVLSATLLYEILLGKATLYAVLVSALVLMAM  VKRKDS
Caphir TRBC3      (D)CGVTS  (A)SYQQGVLSATLLYEILLGKATLYAVLVSALVLMAM  VKRKDS
Caphir TRBC2      (D)CGVTS  (A)SYQQGVLSATLLYEILLGKATLYAVLVSALVLMAM  VKKKDS
Oviari TRBC1      (D)CGVTS  (A)SYQQGVLSATLLYEILLGKATLYAVLVSALVLMAM  VKRKDS
Oviari TRBC3      (D)CGVTS  (A)SYQQGVLSATLLYEILLGKATLYAVPVSALVLMAM  VKRKDS
Oviari TRBC2      (D)CGVTS  (A)SYQQGVLSATLLYEILLGKATLYAVLVSALVLMAM  VKRKDS
Susscr TRBC1      (D)CGFSS  (A)SYQQGVLSATLLYEILLGKAALYAVLVSALVLMAT  VKRKGA
Susscr TRBC3      (D)CGFSS  (A)SYQQGVLSATLLYEILLGKAALYAVLVSALVLMAT  VKRKGA
Susscr TRBC2      (D)CGFSS  (A)SYQQGVLSATLLYEILLGKATLYAVLVSALVLMAT  VKKKDS
Camdro TRBC1      (H)CGFTS  (V)SYQQGVLSATLLYEILLGKATLYAVLVSALVLMAM  VKRKDS
Camdro TRBC3      (D)CGFTS  (V)SYQQGVLSATLLYEILLGKATLYAVLVSALVLMAM  VKRKDS
Camdro TRBC2      (D)CGFTS  (V)SYQQGVLSATLLYEILLGKATLYAVLVSALVLMAM  VRKKDS
                     **.:*     **********:*********:**** ********   *..*.
```
