# Supplementary material for: The expansion of the TRB and TRG genes in domestic goats (Capra hircus) is characteristic of the ruminant species
Source: BMC Genomics. 2020 Sep 11;21:623. doi: 10.1186/s12864-020-07022-x (PMC7488459; doi:10.1186/s12864-020-07022-x)
Supplement: Supplementary file 10 — Additional file 10: Table S4. Correspondence between the germline and the expressed TRBV genes. Description: The nucleotide identity, the Accession number and the IMGT name of each corresponding cDNA clone are reported. [file 12864_2020_7022_MOESM10_ESM.pdf]

Table S4. Correspondence between the germline and the expressed *TRBV* genes.

Clone names	Accession number	IMGT gene	TRBV gene	Nucleotide identity %
TCRVB54	U59406.1	TRBV12S1	TRBV3	99.41
TCRVB21	AF035473.1	TRBV4S1	TRBV4	100
TCRVB49	U59404.1	TRBV4S2	TRBV4	99.42
TCRVB1	U59399.1	TRBV1S3	TRBV5-6	94.56
TCRVB30	AF035476.1	TRBV1S1	TRBV5-8	99.13
TCRVB52	AF035477.1	TRBV1S2	TRBV5-17	97.38
TCRVB17	AF110421.1	TRBV18S1	TRBV5-30	99.05
TCRVB67	U59408.1	TRBV14S1	TRBV6-1	98.84
TCRVB53	U59403.1	TRBV11S1	TRBV7-1	94.86
TCRVB29	AF035475.1	TRBV5S1	TRBV12-2	99.71
TCRVB4	U59405.1	TRBV5S2	TRBV12-1	99.72
TCRVB65	AF035479.1	TRBV7S1	TRBV15	98.84
TCRVB51	U59411.1	TRBV7S2	TRBV15	99.42
TCRVB26	AF035474.1	TRBV2S1	TRBV16	99.12
TCRVB27	U59412.1	TRBV2S2	TRBV16	100
TCRVB90	AF035480.1	TRBV8S1	TRBV19	100
TCRVB19	U59400.1	TRBV9S1	TRBV20	100
TCRVB22	U59407.1	TRBV13S1	TRBV21-6	98.85
TCRVB42	U59409.1	TRBV15S1	TRBV24	98.55
TCRVB12	AF139640.1	TRBV17S1	TRBV25	99.13
TCRVB26	AF139641.1	TRBV17S2	TRBV25	99.13
TCRVB58	U59413.1	TRBV16S1	TRBV26	97.95
TCRVB69	U59401.1	TRBV10S1	TRBV28	98.82
TCRVB59	AF035478.1	TRBV6S1	TRBV29	99.40
TCRVB56	U59402.1	TRBV6S2	TRBV29	100
TCRVB17	AF035472.1	TRBV3S1	TRBV30	98.81
TCRVB33	U59410.1	TRBV3S2	TRBV30	97.13

The nucleotide identity, the Accession number and the IMGT name of each corresponding cDNA clone are reported.
