# Supplementary material for: The expansion of the TRB and TRG genes in domestic goats (Capra hircus) is characteristic of the ruminant species
Source: BMC Genomics. 2020 Sep 11;21:623. doi: 10.1186/s12864-020-07022-x (PMC7488459; doi:10.1186/s12864-020-07022-x)
Supplement: Supplementary file 11 — Additional file 11: Table S5. Description of the related and unrelated TRG genes in the Capra hircus chromosome 4 genome assembly (NCBI Reference Sequence NC_030811.1). The position of all genes and their classification and functionality are reported. [file 12864_2020_7022_MOESM11_ESM.pdf]

Table S5. Description of the related and no related TRG genes in the *Capra hircus* chromosome 4 genome assembly (NCBI Reference Sequence NC_030811.1). The position of all genes and their classification and functionality are reported.

	Gene classification	Functionality	Position[a] (complement)[b]
TRG1	AMPH	F	37658266-37871807
	TRGV11	P[1]	37649887-37650356
	TRGV3-1	F	37637479-37637943
	TRGV3-2	P[2]	37631998-37632424
	TRGV7	F	37626127-37626596
	TRGV10	P[3]	37621823-37622422
	TRGV4	ORF[4]/F[c]	37619358-37619795
	TRGJ5-1	F	37614899-37614951
	TRGJ5-2	ORF	37612359-37612416
	TRGJ5-3	F	37610339-37610388
	TRGC5	P[5]/F[c]	37599987-37607457
	TRGV8	F	37582451-37582920
	TRGV2	F	37568557-37569048
	TRGV9	F	37542570-37543070
	TRGJ3-1	F	37535802-37535861
	TRGJ3-2	P	37531432-37531408
	TRGC3	F	37524473-37530162
	TRGV1	F	37513399-37513883
	TRGJ4-1	F	37507390-37507436
	TRGJ4-2	F	37503996-37504045
	TRGC4	F	37490865-37498645
	LSM8	F	37445643-37454257
TRG2	TRGV5-1	F	70113976-70114468
	TRGJ1-1	F	70176791-70176846
	TRGJ1-2	F	70123417-70123566
	TRGC1	F	70126650-70133540
	TRGV5-2	F	70143121-70143614
	TRGJ2A-1	ORF	70151368-70151427
	TRGJ2A-2	F	70154224-70154273
	TRGC2A	P	70157352-70160366
	TRGV5-3	F	70169850-70170342
	TRGJ2B-1	F	70120816-70120871
	TRGJ2B-2	F	70154224-70154273
	TRGCB2	F	70182576-70191719
	TRGV6	F	70199995-70200469
	TRGJ6-1	F	70206549-70206608
	TRGJ6-2	F	70209834-70209883
	TRGC6	F	70214535-70224555
	STARD3NL	F	70230414-70290261

[a] L-PART1/ V-exon for TRGV genes; [b] for TRG1 genes; [c] F is referred to the same gene detected within CHIR_2.0 assembly

[1] Frameshift in L-PART1; frameshift in V-region

[2] Stop codon in L-PART1; no canonical DONOR-SPLICE site

[3] Stop codon in V-region

[4] No canonical V-HEPTAMER

[5] No stop codon
