# Supplementary material for: The expansion of the TRB and TRG genes in domestic goats (Capra hircus) is characteristic of the ruminant species
Source: BMC Genomics. 2020 Sep 11;21:623. doi: 10.1186/s12864-020-07022-x (PMC7488459; doi:10.1186/s12864-020-07022-x)
Supplement: Supplementary file 13 — Additional file 13: Figure S5. Percent Identity Matrix created by CLUSTAL. Description: Output file obtained by the multiple alignment of the goat and sheep TRGV gene sequences. [file 12864_2020_7022_MOESM13_ESM.pdf]

1: TRGV6_goat	100.00	96.61	38.99	39.78	48.96	49.31	48.23	48.58	43.75	45.61	49.14	49.48	46.38	47.10	46.38	46.38	46.74	41.67	42.01	43.01	42.76	42.31	42.66	45.45	44.36	38.81	39.58
2: TRGV6-1_sheep	96.61	100.00	38.63	39.42	49.31	49.65	48.58	48.94	44.10	45.96	48.45	48.80	46.01	46.74	45.29	46.01	46.38	41.61	42.31	44.06	43.82	44.06	44.41	44.36	43.27	38.46	38.68
3: TRGV11_goat	38.99	38.63	100.00	95.12	44.04	44.04	44.40	43.68	40.42	41.07	43.25	43.01	44.28	43.54	44.28	43.54	42.80	47.04	47.04	50.00	50.18	48.96	50.00	45.29	44.04	48.97	49.31
4: TRGV11-1_sheep	39.78	39.42	95.12	100.00	42.34	42.34	43.43	42.70	38.57	40.07	40.64	40.99	43.28	42.54	43.28	42.54	41.79	46.83	46.83	49.47	49.65	48.42	49.47	45.79	44.89	48.07	48.42
5: TRGV2_goat	48.96	49.31	44.04	42.34	100.00	99.67	85.61	84.91	80.95	82.47	74.57	74.57	74.55	75.63	75.63	75.63	75.99	43.75	43.75	46.02	46.50	46.71	46.02	44.21	43.01	44.98	44.64
6: TRGV2-1_sheep	49.31	49.65	44.04	42.34	99.67	100.00	85.26	84.56	80.61	82.13	74.91	74.91	74.91	75.99	75.99	75.99	76.34	44.10	44.10	46.37	46.85	47.06	46.37	44.21	43.01	45.33	44.98
7: TRGV8_goat	48.23	48.58	44.40	43.43	85.61	85.26	100.00	99.31	84.88	85.07	75.26	74.91	75.27	75.99	75.99	75.99	76.34	43.51	42.46	43.36	43.11	43.36	42.66	42.60	41.37	45.10	45.45
8: TRGV8-1_sheep	48.58	48.94	43.68	42.70	84.91	84.56	99.31	100.00	84.19	84.38	74.57	74.23	74.91	75.63	75.63	75.63	75.99	43.51	42.46	43.01	42.76	43.01	42.31	42.24	41.01	44.76	45.10
9: TRGV9_goat	43.75	44.10	40.42	38.57	80.95	80.61	84.88	84.19	100.00	96.63	70.67	70.37	72.28	73.33	72.63	72.63	72.98	38.78	39.46	40.68	40.75	40.68	40.00	39.86	39.72	45.12	45.45
10: TRGV9-1_sheep	45.61	45.96	41.07	40.07	82.47	82.13	85.07	84.38	96.63	100.00	71.77	71.77	73.76	74.82	74.11	74.11	74.47	39.18	39.86	41.44	42.21	42.12	41.44	41.70	41.20	46.58	46.92
11: TRGV1_goat	49.14	48.45	43.25	40.64	74.57	74.91	75.26	74.57	70.67	71.77	100.00	98.67	78.95	80.35	79.30	79.65	81.05	43.88	44.56	42.71	43.84	43.05	43.05	39.58	38.38	43.10	44.11
12: TRGV1_sheep	49.48	48.80	43.01	40.99	74.57	74.91	74.91	74.23	70.37	71.77	98.67	100.00	78.25	79.65	78.60	78.95	80.35	42.52	43.20	43.05	44.18	43.39	43.39	39.22	38.03	43.05	44.07
13: TRGV5-1_sheep	46.38	46.01	44.28	43.28	74.55	74.91	75.27	74.91	72.28	73.76	78.95	78.25	100.00	97.54	95.44	97.19	96.14	43.73	44.09	42.50	42.24	42.86	42.86	40.59	40.81	45.36	46.43
14: TRGV5-2_sheep	47.10	46.74	43.54	42.54	75.63	75.99	75.99	75.63	73.33	74.82	80.35	79.65	97.54	100.00	97.19	97.54	97.89	43.01	43.37	42.14	42.60	42.50	42.50	40.96	40.44	45.36	46.43
15: TRGV5-2_goat	46.38	45.29	44.28	43.28	75.63	75.99	75.99	75.63	72.63	74.11	79.30	78.60	95.44	97.19	100.00	97.54	97.89	41.22	41.58	42.14	41.88	42.50	41.79	40.96	39.71	45.71	46.43
16: TRGV5-1_goat	46.38	46.01	43.54	42.54	75.63	75.99	75.99	75.63	72.63	74.11	79.65	78.95	97.19	97.54	97.54	100.00	98.25	42.65	43.01	42.50	42.96	42.86	42.86	40.59	40.07	45.36	46.43
17: TRGV5-3_goat	46.74	46.38	42.80	41.79	75.99	76.34	76.34	75.99	72.98	74.47	81.05	80.35	96.14	97.89	97.89	98.25	100.00	41.58	41.94	41.07	41.52	41.43	41.43	41.33	40.07	45.36	46.43
18: TRGV7_goat	41.67	41.61	47.04	46.83	43.75	44.10	43.51	43.51	38.78	39.18	43.88	42.52	43.73	43.01	41.22	42.65	41.58	100.00	98.37	59.47	61.41	60.13	61.13	45.67	45.86	50.50	49.50
19: TRGV7_sheep	42.01	42.31	47.04	46.83	43.75	44.10	42.46	42.46	39.46	39.86	44.56	43.20	44.09	43.37	41.58	43.01	41.94	98.37	100.00	59.47	61.41	60.13	61.13	44.98	45.17	49.83	48.84
20: TRGV3-2_goat	43.01	44.06	50.00	49.47	46.02	46.37	43.36	43.01	40.68	41.44	42.71	43.05	42.50	42.14	42.14	42.50	41.07	59.47	59.47	100.00	95.68	95.39	96.38	45.02	45.21	47.68	48.34
21: TRGV3-2_sheep	42.76	43.82	50.18	49.65	46.50	46.85	43.11	42.76	40.75	42.21	43.84	44.18	42.24	42.60	41.88	42.96	41.52	61.41	61.41	95.68	100.00	96.01	97.67	46.53	46.71	48.83	49.50
22: TRGV3-1_goat	42.31	44.06	48.96	48.42	46.71	47.06	43.36	43.01	40.68	42.12	43.05	43.39	42.86	42.50	42.50	42.86	41.43	60.13	60.13	95.39	96.01	100.00	97.70	44.33	44.52	48.34	49.01
23: TRGV3-1_sheep	42.66	44.41	50.00	49.47	46.02	46.37	42.66	42.31	40.00	41.44	43.05	43.39	42.86	42.50	41.79	42.86	41.43	61.13	61.13	96.38	97.67	97.70	100.00	45.36	45.55	48.68	49.34
24: TRGV4_goat	45.45	44.36	45.29	45.79	44.21	44.21	42.60	42.24	39.86	41.70	39.58	39.22	40.59	40.96	40.96	40.59	41.33	45.67	44.98	45.02	46.53	44.33	45.36	100.00	97.95	47.93	48.28
25: TRGV4_sheep	44.36	43.27	44.04	44.89	43.01	43.01	41.37	41.01	39.72	41.20	38.38	38.03	40.81	40.44	39.71	40.07	40.07	45.86	45.17	45.21	46.71	44.52	45.55	97.95	100.00	48.11	48.11
26: TRGV10_goat	38.81	38.46	48.97	48.07	44.98	45.33	45.10	44.76	45.12	46.58	43.10	43.05	45.36	45.36	45.71	45.36	45.36	50.50	49.83	47.68	48.83	48.34	48.68	47.93	48.11	100.00	96.42
27: TRGV10-1_sheep	39.58	38.68	49.31	48.42	44.64	44.98	45.45	45.10	45.45	46.92	44.11	44.07	46.43	46.43	46.43	46.43	46.43	49.50	48.84	48.34	49.50	49.01	49.34	48.28	48.11	96.42	100.00
