# Supplementary material for: The expansion of the TRB and TRG genes in domestic goats (Capra hircus) is characteristic of the ruminant species
Source: BMC Genomics. 2020 Sep 11;21:623. doi: 10.1186/s12864-020-07022-x (PMC7488459; doi:10.1186/s12864-020-07022-x)
Supplement: Supplementary file 14 — Additional file 14: Figure S6. (A) Description of the goat TRGV genes. Description: The IMGT Protein display of the goat TRGV genes compared with the sheep orthologous genes. The deduced amino acid sequences of the TRGV genes were manually aligned according to IMGT unique numbering for the V-REGION [33] to maximize homology. Only functional genes, ORF and in-frame pseudogenes are shown. All sequences exhibit the typical framework regions (FR) and complementarity determining regions (CDR) and the four amino acids (indicated in bold): cysteine 23 (1st-CYS) in FR1-IMGT, tryptophan 41 (CONSERVED-TRP) in FR2-IMGT, with the exception of TRGV1 and TRGV5 subgroups, hydrophobic (here L, F and M) 89, and cysteine 104 (2nd-CYS) in FR3-IMGT. Conversely, CDR-IMGT vary in amino acid composition and length. The description of the strands and loops and of the FR-IMGT and CDR-IMGT is according to the IMGT unique numbering for V-REGION [33]. [file 12864_2020_7022_MOESM14_ESM.pdf]

(A)

Gene	Functionality	LEADER	FR1-IMGT (1-26): A (1-15), B (16-26)	CDR1-IMGT (27-38): BC (27-38)	FR2-IMGT (39-55): C (39-46)	FR2-IMGT: C' (47-55)	CDR2-IMGT (56-65): C'C" (56-65)	FR3-IMGT (66-104): C" (66-74)	FR3-IMGT: D (75-84)	FR3-IMGT: E (85-96)	FR3-IMGT: F (97-104)	CDR3-IMGT (105-117): FG (105-117)
			1 10 15 16 23 26	27 38	39 41 46	47 55	56 65	66 74	75 80 84	85 89 96	97 104	105
TRGV1												
Caphir	F	MLWALVLPLVFLALVTQV	SSNAKGAQMSVTGKT..WETTSFTCDFT	QDV.......KY	IHLYKQQE	GMAPRRLFY	YDVY..YSKI	EFESGVNKA	KYSVYKGAAG	RSYRFAILNLEH	SDSGTYYC	AVWDK..
Oviari	F	MLWALVLPLVFLALVTQV	SSNAKGAQMSVTAKT..WDTTSFTCDFT	QDV.......KY	IHLYKQQE	GMAPRRLFY	YDVY..YSKI	EFESGIDKA	KYSVYKGAAG	RSYRFAILNLEH	SDSGTYYC	AVWDK
TRGV2												
Caphir	F	MRVPALLLVFLAPVMQV	SSNMEGEKMSITKAAVPGAFVEIICDLI	TQT......VKY	IHWYKYQE	GTAPRRLLY	YDIS..YSKI	VLESGISEG	KYKVYK...E	KSYTFAISNLQE	SDSGMYYC	AVWEK..
Oviari	F	MRVPALLLVFLAPVMQV	SSNMEGEKMSITKAAVPGAFVEIICDLI	TQT......VKY	IHWYKHQE	GTAPRRLLY	YDIS..YSKI	VLESGISEG	KYKVYK...E	KSYTFAISNLQE	SDSGMYYC	AVWEK..
TRGV3-1												
Caphir	F	MSPLEAFTFFSFWASGLG	LSKVEQAQISLSTEV..KKSIDIHCKIE	STNF....ESET	VYWYRQKR	NQALEHLVY	VIST...TTA	ARNQVDGKN	KIEARKDARM	FTSTLTVNFVEK	DDVGIYYC	AGWS...
Oviari	F	MSPLEAFTFFSFWASGLG	LSKVEQAQISLSTEV..KKSIDIHCKIE	STDF....ESEA	VYWYRQKR	NQALEHLVY	VIST...TTA	ARNQVDGKN	KIEARKDARM	FTSTLTVNFVEK	EDVGIYYC	AGWS...
TRGV3-2												
Caphir	P	MSPLEAFTFHFR*MTGLG	LSKVEQAQISLSTEV..KKSIDIHCKTE	STNF....ESET	VYWYWQKR	NQALEHLVY	VIST...TTA	ARNQVDGKN	KIEARKDARM	FTSTLTVNFVEK	EDVGIYCC	AGWD...
Oviari	P	MSPLEAFTFFSFWASGLG	LSKVEQAQISLSTEV..KKSIDIHCKIE	STDF....ESET	VYWYRQKR	NQALEHLVY	VIST...TTA	ARNQVDGKN	KIEARKDAQM	FASTLMVNFVEK	EDVGIYYC	AGWD...
TRGV4												
Caphir	ORF	MPTTWIFLMSLAWVYGDI	KMKISQHQLSSTRRP..DRTVHISCKLS	GVPL....ENAI	VHWYQEKE	GEPLKRILY	GS......AN	SYKLDKPNS	RLEMDNK.KN	GIFYLVINNVVK	SDEATYYC	ACWD...
Oviari	F	MPTTWIFLMSLAWVYGDI	KMRISQNQLSSTRRP..DRTVHISCKLS	GVPL....ENAI	VHWYQEKE	GEPLKRILY	GS......AN	SYKLDKPNS	RLEMDNK.KN	GIFYLIINNVVK	SDEATYYC	ACWD...
TRGV5-1												
Caphir	F	MRAPALLLVVLAPVMQV	SSNL....MLITSKT..GETASFPCDLT	QGA.......TY	IHLYKHEE	GMAPRRLLY	YDSY..NSKP	VLESGISGT	KYHVYKG.TG	MSYTFTIVNLQA	SDSGIYYC	AIWDK..
Oviari	F	MRAPALLLVVLAPVMQV	SSNL....MLITRKT..GETASFPCDLT	HGA.......IY	IHLYKHEE	GMAPRRLLY	YDSY..NSKP	VFESGISGT	KYHVYKG.TG	SSYTFTIVNLQA	SDSGIYYC	AIWDK..
TRGV5-2												
Caphir	F	MRAPALLLVVLAPVMQV	SSNL....MLITSKT..GETASFPCDLT	RRA.......TY	IHLYKHEE	GMAPRRLLY	YHSY..NSKP	VLESGISGT	KYHVYKG.TG	RSYTFAIQNLQA	SDSGIYYC	AIWDK..
Oviari	F	MRAPALLLVVLAPIMQV	SSNL....MLITRKT..GETASFPCDLT	QGA.......TY	IHLYKHEE	GMAPRRLLY	YDSY..NSKP	VFESGISGT	KYHVYKG.TG	RSYTFAILNLQA	SDSGIYYC	AIWDK..
TRGV5-3												
Caphir	F	MRAPALLLVVLAPVMQV	SSNL....MLITSKT..GETASFPCDLT	QGA.......TY	IHLYKHEE	GMAPRRLLY	YDSY..NSKP	VLESGISGA	KYHVYKG TG	RSYTFAILNLQA	SDSGIYYC	AIWDK..
TRGV6												
Caphir	F	MGSSLGAEGRAVLGRLALLWALVVPGIQQ	EIRLSQRSVMVGSAG...GAMTMPCQVS	RSV.......NY	VHWFRQLE	GQAPERLLY	LALS..KRDV	QWDSVLGGD	KVSAARGGDG	KSCTMSLRKLAK	SDEGLYYC	AAWDP..
Oviari	F	MGSSLGAEGRAVLGRLTLLWALVVPGIQQ	EIRLSQRSVMVGSAG...GAMTMPCSVS	KSV.......DY	VHWFRQLE	GQAPERLLY	LALS..KRDV	QWDSVLGGD	KVSAARGGDG	KSCTMSLRKLAK	SDEGLYYC	AAWGS..
TRGV7												
Caphir	F	MALLEAVLFSSLWSFGLG	QLTLEQPELSVTGTR..EKSIIMTCKVF	SKDF....SKDY	IHWYRQKP	DQGLEQLLY	VST....APA	QNHLGGKKN	KLEARKDAPS	STSTLKISFLEK	EDEATYYC	AGWLST.
Oviari	F	MALLEAVLFSSLWSFGLG	QLTLEQPELSVTGTR..EKSIIMTCKVF	SKDF....SKDY	IHWYRQKP	DQGLEQLLY	VST....APA	QNHLGGKKN	KLEARKDAPS	STSTLKISFLEK	EDEATYYC	AGWLSA.
TRGV8												
Caphir	F	MLWVPALLLIFLAPVTQV	SSNMEEDKLSVTRAT..GSSVVIDCDLT	QN.........Y	IHWYKFQE	GTVPRRLLY	YDVY..YSKV	VLESGISEG	KYHVYKG.TG	KSYTFVISNLQE	SDSGTYYC	AVWEK..
Oviari	F	MLWVPA.LLIFLAPVTQV	SSNMGEDKLSVTRAT..GSSVVIDCDLT	QN.........Y	IHWYKFQE	GTVPRRLLY	YDVY..YSKV	VLDSGISEG	KYHVYKG.TG	KSYTFVISNLQE	SDSGTYYC	AVWEK..
TRGV9												
Caphir	F	MLRAPVLLLVFLALVTQV	SSNMEGNRMSITRAI..GSSAVIPCDLP	TQN......IKY	IHWYKFQE	GTIPRRLLY	YDVS..YSKV	VLESGISPG	KYHCYEG.TD	KIYKFVISSLQE	SDSGVYRC	AVWEK..
Oviari	F	MLRAPVLLLVFLALVTQV	SSNMEGDRMSITRAA..GSSAVIPCDLP	TQN......IKY	IHWYKFQE	G.IPRRLLY	YDVS..YSKV	VLESGISPG	KYHCYEG.TD	KIYKFVISSLQE	SDSGVYHC	AVWEK..
TRGV10												
Caphir	P	MLLLPQVLVVASLWTYTSG	DLPITQRITSITKKK..GNMAFLECQIK	IDKLK...KNVY	MHWYRQKP	EQPLKRILY	ISSN...ENV	IHEQGISEE	RYEARKWPSN	ALVSLRIH*ATE	EEAGLYYC	ACWLG..
