# Supplementary material for: The expansion of the TRB and TRG genes in domestic goats (Capra hircus) is characteristic of the ruminant species
Source: BMC Genomics. 2020 Sep 11;21:623. doi: 10.1186/s12864-020-07022-x (PMC7488459; doi:10.1186/s12864-020-07022-x)
Supplement: Supplementary file 15 — Additional file 15: Figure S6. (B) Description of the goat TRGJ genes. Description: Nucleotide and deduced amino acid sequences of the TRBJ genes. The consensus sequence of the heptamer and nonamer is provided at the top of the figure and is underlined. The numbering adopted for the gene classification is reported on the left of each gene. The donor splice site for each TRBJ is shown. The canonical FGXG amino acid motifs are underlined. The TRGJ3–2 pseudogene and the TRGJ5–2 and TRGJ7–1 ORF are indicated in italics. [file 12864_2020_7022_MOESM15_ESM.pdf]

(B)

TRGJ gene name	J-NONAMER GGTTTTTGT	J-SPACER ************	J-HEPTAMER CACTGTG	J-REGION	5'splice donor
TRGJ1-1	gattattgt	aggagcatttat	cattgtg	ACAGCTCGGGCTGGAAGAAGATATTTGGAGAAGGAGCTAACATCATAGTAACTCCTTCCG<br>S S G W K K I F G E G A N I I V T P S	gtaagt
TRGJ1-2	agtttttca	tacagcttgaat	cattgtg	GAATAATTACTTAAAAAACTTCAAAGTTGGAGCAAAACTCATTGTCACAG<br>N N Y L K N F K V G A K L I V T	gtaggt
TRGJ2A-1	*gattattgt*	*cggagcatttat*	*cattgtg*	*ACAGCTCGGGCTGGAAGAAGATATTTGGAGAAGAAGCTAACATCATAGTAACTCCTCCCG*<br>*S S G W K K I F G E E A N I I V T P P*	*gtaagt*
TRGJ2A-2	agtttttga	tatgacttgaat	cactgtg	GAATAATTACATAAAAAACTTCAATGTTGGAGCAAAACTCATTGTCACAG<br>N N Y I K N F N V G A K L I V T	gtaggt
TRGJ2B-1	gattattgt	aggagcatttat	cattgtg	ACAGCTCGGGCTGGAAGAAGATATTTGGAGAAGGAGCTAACATCATAGTAACTCCTCCCA<br>S S G W K K I F G E G A N I I V T P P	gtaagt
TRGJ2B-2	agtttttga	tatgacttgaat	cactgtg	GAATAATTACATAAAAAACTTCAATGTTGGAGCAAAACTCATTGTCACAG<br>N N Y I K N F N V G A K L I V T	gtaggt
TRGJ3-1	gattattgt	aggagcttcaac	cagtgtg	ACAGTGCAGGCTGGAAGAAGCTATTTGGAAAAGCAACTGAGCTCATAGTAGCTTCCCCTG<br>S A G W K K L F G K A T E L I V A S P	gtgagt
TRGJ3-2	*cgatttcta*	*tatgagttgaat*	*aattttg*	*AAATTATTATAGAAAAATGTTTGTCAATGGAATAAAATTTTTGTCATAG*<br>*N Y Y R K M F V N G I K F L S **	*gtatat*
TRGJ4-1	gcttcaacc	agggtaatggct	cagaatg	AAAGAAGATATTTGGAAAAGGACCTAAGCTCATAATAGCTCCCCATG<br>K K I F G K G P K L I I A P H	gtaagt
TRGJ4-2	agtttttga	tacaggctgaac	cactgtg	TAATAGTTACATAAAAAACTTCAACGTTGGAACAAAGCTTGTTGTCACAG<br>N S Y I K N F N V G T K L V V T	gtaggt
TRGJ5-1	gatttttgt	agaagctccaat	cattgtg	ACAAGGGATCAAGGTATTTGGTGAAGGAACTAAGCTCGTAGTAATACCTCCTG<br>Q G I K V F G E G T K L V V I P P	gtaagt
TRGJ5-2	*gatttctat*	*aaaagattttta*	*ggtagtg*	*CAAACAGGTGGCCAGAGCATCAAAGTGTTTGGTTCCAGAACACAACTTATTGTTACAG*<br>*Q T G G Q S I K V F G S R T Q L I V T*	*gtgaag*
TRGJ5-3	agtttttga	tatgggttgaat	cactgtg	GAGTTATTATGTAAAAATCTTCGGCGATGGGACAAAACTTGTAGTCACAG<br>S Y Y V K I F G D G T K L V V T	gtgggt
TRGJ6-1	agctcactg	taggagcctgaa	cagtgtg	ACAGCTCAGGTTGGAAGAAGATGTTTGGAGAAGGAACTGAGCTCATAGTAGCTCCCTGTG<br>S S G W K K M F G E G T E L I V A P C	gtaagt
TRGJ6-2	agtttttgc	cacaggttgaat	cactgtg	GAATTTAAACAACATGATCTTTGGTGGAGGAACAAAAGTTTTCGTCCAGG<br>N L N N M I F G G G T K V F V Q	gtaagt
