# Supplementary material for: The expansion of the TRB and TRG genes in domestic goats (Capra hircus) is characteristic of the ruminant species
Source: BMC Genomics. 2020 Sep 11;21:623. doi: 10.1186/s12864-020-07022-x (PMC7488459; doi:10.1186/s12864-020-07022-x)
Supplement: Supplementary file 16 — Additional file 16: Figure S6. (C) Description of the goat TRGC genes. Description: The IMGT Protein display of the goat TRGC gene compared with the sheep Cγ proteins. The descriptions of the strands and loops were collected according to the IMGT unique numbering for the C-DOMAIN [54]. [file 12864_2020_7022_MOESM16_ESM.pdf]

(C)

```
                               A        AB      B           BC        C     CD      D          DE           E      EF   F         FG          G
                             (1-15)          (16-26)     (27-38)   (39-45)       (77-84)                 (85-96)     (97-104)  (105-117)   (118-128)
                         --------------->  ----------->            ------->      -------->             ------------> -------->            ----------
                         1        10  15   16    23 26 27      38 3941 45       77    84              85  89    96  97   104 105       117 118
                 87654321|........|....|123|......|..|  |.........|  |.|...|1234567|......|12345677654321|...|......|12|......|  |...........|  |........
Caphir TRGC1   (E)RNLAADTSPKPTVFLPSIAEINH..DNAGTYLCLLE NFFP..DVIT VSWRVKNDKRAL..PSQQGNTMKTKD.......TYMKLSWLTVTEN....SMDKQHVC VVKHQKNIGGIDQ EIIFPSIKE
Oviari TRGC1   (E)RNLAADTSPKPTVFLPSIAEINH..DNAGTYLCLLE KFFP..DVIT VSWRVKNDKRAL..PSQQGNTMKTKD.......TYMKLSWLTVTEN....SMDKQHVC VVKHQKNIGGIDQ EIIFPSIKE
Caphir TRGC2B  (E)RNLAADTSPKPTVFLPSIAEINH..DNAGTYLCLLE NFFP..DVIT VSWRVKNDKRAL..PSQQGNTMKTKD.......TYMKLSWLTVTEN....SMDKQHVC VVKHQKNIGGIDQ EIIFPSIKE
Oviari TRGC2   (E)RNLAADTSPKPTVFLPSIAEINH..DNAGTYLCLLE KFFP..DVIT VSWRVKNDKRAL..PSQQGNTMKTKD.......TYMKLSWLTVTEN....SMDKQHVC VVKHQKNIGGIDQ EIIFPSIKE
Caphir TRGC3   (D)RDLDKDMSPKPTMFLPSITEIKR..DNNGTYLCLLE DFFP..HVIK VYWREKRGNKVL..PSQEGKTIKTDD.......TYMKLSWLTVSGN....SMDKEHMC IVKHEKNKRGTNQ EILFPAVNE
Oviari TRGC3   (D)RDLDIDMSPKPTMFLPSITEIKR..DNSGTYLCLLE DFFP..HVIK VYWREKRGNKVL..PSQEGKTIKTLD.......TYMKFSWLTVSGN....SMDKEHMC IVKHEKNKRGTNQ EILFPAVNE
Caphir TRGC4   (D)RNLATDLSPKPTIFLPSIAEINH..NKTGTYLCLLE KFFP..DIIK VYWKEKDGNRAL..PSQQGNTMNTTD.......TYMKLSWLTVTEN....SMDKEHIC VVQHERNIRGINQ EILFPSINE
Oviari TRGC4   (N)RNLATDLSPKPIIFLPSIAEINH..SKTGTYLCLLE KFFP..DIIK VYWKEKDGNRAL..PSQQGNTMNTTD.......TYMKLSWLTVTEN....SMDKEHIC VVQHERNIRGINQ EILFPSINE
Caphir TRGC5   (D)RRLDGDLFPKPTIFFPSVEEVKR..HSAGTHLCLLQ NFFP..DAIK VQWKEKNGNTIL..ESHQGNIIKTND.......TYMKFSWLTLTKK....AMEKEHVC IVKHENNKGGRDQ EILFSPVNK
Oviari TRGC5   (D)RRLDGDLFPKPTIFFPSVEEVKR..HSAGTHLCLLQ NFFP..DAIK VQWKEKNGNTIL..ESHQGNIIKTND.......TYMKFSWLTLTKK....AMGKEHVC IVKHENNKGGRDQ EILFSPVNK
Caphir TRGC6   (D)KNLPTDIIPKPTIFLPSINEVNH..QQTATYLCLLE NFFP..DVIK VSWKEKNGNRVL..PSQQGNTMKTNN.......TYMKFSWLTVTEN....SMKKEHMC IVRLEKNAGGKDQ EILFPAVNE
Oviari TRGC6   (D)KNLPTDIIPKPTIFLPSINEVNH..QQTATYLCLLE NFFP..DVIK VSWKEKNGKRVL..PSQQGNTMKTNN.......TYMKFSWLTVTED....SMKKEHMC IVRLEKNAGGKDQ EILFPAVNV
```

```
                                         CONNECTING-REGION                                                  |   TRANSMEMBRANE-REGION  |CYTOPLASMIC-REGION
                     [EX2A]                            [EX2B]              [EX2C]                                [EX3]
Caphir TRGC1    (V)VTSAVT.....TTEPPTTEPPNDCLTDES                                              (S)ALQLQLTTTSAYYTYLLLLLLSAVYFVVIISCVFRRTGVCCDGKIS
Oviari TRGC1    (V)VTSAVT..........TTKPPNDCLTDES                                              (S)ALRLQLTTTSAYNTYLLLLLLSTVYFVVIISCVFRRTGVWSDWKIS
Caphir TRGC2B   (V)VSSAVTTTEPPTTEPPTTEPPNDCLTDES  (K)VTGTGSKKACLKDES  (E)VTGDT.NSTKARLEGEN  (S)ALQLQLTTTSAYYTYLLLLLRSAVYFVVIISCVFRRTGVCCDGKI.
Oviari TRGC2    (V)VTSAVTTTEPPTTEPPTTEPPNDCLTDES  (K)VTGTGSKTACLKDER  (E)VTGDT.NSTKACQEGES  (S)ALQLQLMNTSAYYTYLLLLLLSTVYFVVIISCVFRRTGICCDGKIS
Caphir TRGC3    (V)VSSVVT..........TTKPPNDGLKDRS                      (E)KQVPVANSTKACLKDEN  (N)TLQLQLMNTSAYYTYLLLLIKSTVYFAIITSCVFRRTGVCGNQKSS
Oviari TRGC3    (V)VSSVVA..........TTKPPNDGLKDKS                      (E)KQVPVANSTKACLKDEN  (N)TLQLQLMSTSAYYTYLLLLIKSTVYFAIITSCVFRRTGVCGNQKSS
Caphir TRGC4    (V)VSSIVP..........TTEAPSDCLNQES  (K)VTGTGSKKVCLKDES  (E)VTADN.NSTKVCLEDES  (N)TLQLQLMNTSAYYTYLLLLLKSVVYFIIITSCVFRRTGICCDGKNS
Oviari TRGC4    (V)VSSIVP..........TTESPSDCLNQES  (K)VTGTGSKKACLKDES  (E)VTADN.NSTKVCLEDES  (N)TLQLQLMNTSAYYTYLLLLLKSVVYFIIITSCVFRRTGMCCDGKNL
Caphir TRGC5    (E)VAT..................RACMKKES                                              (D)TLQLQFASTSAYYTYLLLLLKSMIYFSIIAFCVFWRTGIFSNGKIF
Oviari TRGC5    (E)VAT..................HACMKKES                                              (D)TLQLQFASTSAYYTYLLLLLKSMIYFSIIAFCVFWRIGIFSNGKIF
Caphir TRGC6    (V)FSPVVA..........TTGPPDDCLQDES  (E)VTDTDFTKVCSRGES  (E)VN....NSTKACLKDKN  (N)TVELQLTYNSAYYTYLLLLLKSAVYFVTTFCCVFRRTGVCRDGKSS
Oviari TRGC6    (V)FSPVVA..........TTEPPDDCLQDEI  (E)VTDTDFTKVCSRGEI  (E)VT....DSTKACLKDEI  (D)TVELQLAYNSAYYTYLLLLLKSAIYFVTSSCCVFRRTGVCHDGKSS
```
